# Rational flux-tuning of *Halomonas bluephagenesis* for co-production of bioplastic PHB and ectoine

**DOI:** 10.1038/s41467-020-17223-3

**Published:** 2020-07-03

**Authors:** Hong Ma, Yiqing Zhao, Wuzhe Huang, Lizhan Zhang, Fuqing Wu, Jianwen Ye, Guo-Qiang Chen

**Affiliations:** 1 Center for Synthetic and Systems Biology, School of Life Sciences, Tsinghua University, Beijing, 100084 China; 2 MOE Key Lab of Industrial Biocatalysis, Department of Chemical Engineering, Tsinghua University, Beijing, 100084 China; 3 Tsinghua-Peking Center for Life Sciences, School of Life Sciences, Tsinghua University, Beijing, 100084 China

**Keywords:** Metabolic engineering, Applied microbiology, Biopolymers

## Abstract

Ectoine, a compatible solute synthesized by many halophiles for hypersalinity resistance, has been successfully produced by metabolically engineered *Halomonas bluephagenesis*, which is a bioplastic poly(3-hydroxybutyrate) producer allowing open unsterile and continuous conditions. Here we report a de novo synthesis pathway for ectoine constructed into the chromosome of *H. bluephagenesis* utilizing two inducible systems, which serve to fine-tune the transcription levels of three clusters related to ectoine synthesis, including *ectABC*, *lysC* and *asd* based on a GFP-mediated transcriptional tuning approach. Combined with bypasses deletion, the resulting recombinant *H. bluephagenesis* TD-ADEL-58 is able to produce 28 g L^−1^ ectoine during a 28 h fed-batch growth process. Co-production of ectoine and PHB is achieved to 8 g L^−1^ ectoine and 32 g L^−1^ dry cell mass containing 75% PHB after a 44 h growth. *H. bluephagenesis* demonstrates to be a suitable co-production chassis for polyhydroxyalkanoates and non-polymer chemicals such as ectoine.

## Abstract (web summary)

*Halomonas bluephagenesis* is a halophilic platform bacterium for next generation industrial biotechnology. Here, the authors employ a stimulus response-based flux-tuning method for coproduction of bioplastic PHB and ectoine under open unsterile and continuous growth conditions.

## Introduction

Ectoine (1,4,5,6-tetrahydro-2-methyl-4-pyrimidinecarboxylic acid) is one of the best well-known compatible solutes produced by halophiles for hypersalinity resistance^1^. Ectoine was reported to show protections for living cells by enhancing the stabilities of proteins, nucleic acids, and cell membranes, enabling survival of halophiles under the circumstances of hypersalinity, high temperatures, extreme pH and exposure to chemical agents^2–7^. Therefore, ectoine has been exploited for commercial applications as an ingredient for cosmetics^8^, medicines^9^, and organ transplantation maintenance^10^. However, the high production cost is still unacceptable for wider applications^11^.

Commercially, fermentative production of ectoine by microorganisms under sterile bioprocesses is the major solution to meet its increasing demands^12^. The major biosynthesis pathway of ectoine containing its operon *ectABC* encoding l-2,4-diaminobutyrate acetyltransferase (*ectA*), l-2,4-diaminobutyrate transaminase (*ectB*) and ectoine synthase (*ectC*), respectively, has been clearly characterized to be a highly conservative cluster across halophile species, allowing three-step biocatalysis to form ectoine from l-aspartate-β-semialdehyde (ASA), an important intermediate involved in several amino acids synthesis, such as threonine and lysine^13–15^. Interestingly, many halophiles also contain ectoine hydrolase encoded by *ectD* for converting ectoine into 5-hydroxyectoine as both carbon- and nitrogen-source used under nutrient-limited conditions, which is a dominating bypass limiting the high-level intracellular accumulation of ectoine^16,17^.

Recent progresses have exemplified the enhanced production of ectoine by recombinant halophiles, halophilic archaea, *E. coli* and *Corynebacterium glutamicum* employing various synthetic biology strategies. Specifically, halophiles were first grown in media with a high salt concentration for sufficient ectoine synthesis, subsequently cells were quickly transferred into desalted water for effective secretion of ectoine into extracellular space, termed ‘bacterial milking’^18,19^. Interestingly, it was reported that the deletion of TeaABC transporter in *Halomonas elongata* DSM 2581 led to an efficient secretion of ectoine in extracellular space during a high-cell-density fermentation^20,21^. Additionally, the heterologous expression of *ectABC* cluster from halophiles by engineered model microorganisms enabling sufficient ectoine biosynthesis has been achieved: *ectABC* from *Chromohalobacter salexigens* and *Halomonas elongata* DSM 2581, the most studied cluster, were overexpressed in *E. coli* for enhanced production of ectoine from l-aspartate and glucose^14,22^. Remarkably, metabolically engineered *E. coli* W3110 and *C. glutamicum* with bypass deletion, transcriptional (promoter) and translational (RBS) tuning based on high-throughput combinatory constructs and bioprocess optimizations, were successfully used to direct metabolic fluxes towards ectoine synthesis from glucose for efficient ectoine synthesis^6,23^.

Compared with current industrial biotechnology (CIB) based on model microorganisms, next generation industrial biotechnology (NGIB) based on *Halomonas bluephagenesis* TD01, a natural poly(3-hydroxybutyrate) (PHB) producer, has been successfully developed for achieving cost-effective production under open unsterile and continuous growth conditions^24–27^. PHB, a family member of microbial biopolyesters synthesized by many bacteria and archaea including *E. coli*, *Pseudomonas putida* and *Cupriavidus necator* et al., is a model biodegradable polymer for commercial applications^28–32^*. H. bluephagenesis* has been successfully engineered to produce cost-effective PHB^33–35^ from lab-scale to pilot-scale (5000-L) fermentation^27^. It has become highly desirable to engineer *H. bluephagenesis* as a versatile chassis for productions of valuable chemicals at a competitive cost, or for co-production of PHA (polyhydroxyalkanoates, or PHB) with a small molecular chemical to increase the process economy^36,37^.

*H. bluephagenesis* could be a suitable host to engineer for ectoine production due to its fast growth and high PHB accumulation under high-salt conditions. So far *H. bluephagenesis* has been manipulated via various approaches including CRISPR/Cas9^34^, stimulus response-based flux-tuning (SR-FT)^38^, genomic integrations, inducible promoter mining^35^, and promoter engineering^39^. These concreted efforts enable rational strain engineering of complexity for synthesizing diverse metabolic targets, strongly expanding the capability of *H. bluephagenesis* as a versatile production chassis. Generally, static optimization is a widely used strategy to fine-tune the pathways involved in multi-gene regulation largely dependent on high throughput constructs and machine learning-based analysis^40–42^, leading to high cost of clone construction with a long R&D cycle.

**Fig. 1:**
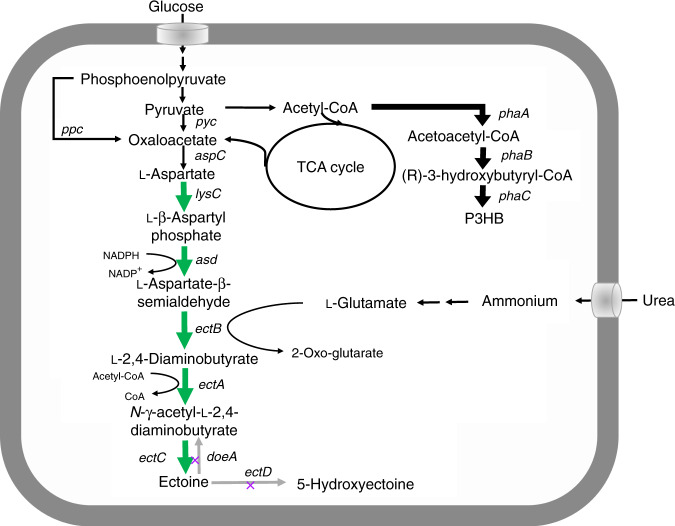
Metabolic engineering for ectoine and PHB co-production by *H. bluephagenesis* TD1.0. Endogenous metabolic fluxes of high efficiency involved in PHB synthesis are shown in black bold lines. Fine-tuned ectoine biosynthesis pathway for enhanced production is shown in green bold lines. Genes *doeA* and *ectD* putatively encoding ectoine hydrolase and ectoine hydroxylase, respectively, are deleted to reduce ectoine degradation. *phaA*, 3-ketothiolase; *phaB*, NADPH (or NADH)-dependent acetoacetyl-CoA reductase; *phaC*, PHA synthase; *ectA*, l-2,4-diaminobutyrate acetyltransferase; *ectB*, l-2,4-diaminobutyrate transaminase; *ectC*, ectoine synthase; *asd* and *lysC* encoding l-aspartate-semialdehyde-dehydrogenase and aspartokinase, respectively, are cloned from *Corynebacterium glutamicum*.

This study aims to chromosomally engineer *H. bluephagenesis* for enhanced production of ectoine from glucose using “stimulus response-based flux-tuning” method (Fig. 1). Specifically, the ectoine titer reaches 6.3 and 28 g L^−1^ during a 48 h growth in 500 mL shake flask and 28 h fed-batch cultivation in 7-L bioreactor, respectively, by chromosomally engineered *H. bluephagenesis* TD-ADEL-58 harboring fine-tuned ectoine synthesis pathway. At the same time, co-production of ectoine and PHB is also studied for possible economic improvement^43–45^, which results in 8 g L^−1^ ectoine and 32 g L^−1^ dry cell mass (DCM) containing 75% PHB after 44 h fed-batch fermentation.

## Results

### Identification of ectoine synthesis pathway

**Fig. 2:**
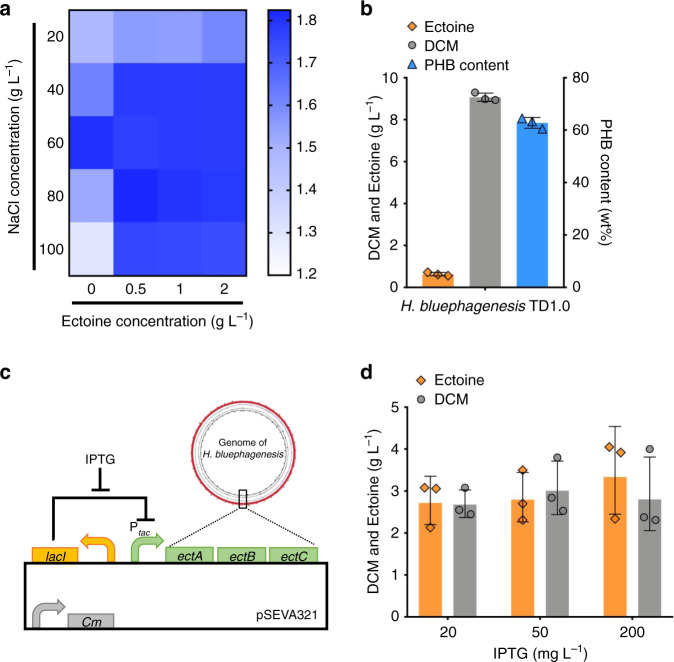
Identification of the ectoine biosynthesis pathway of *H. bluephagenesis* TD1.0. **a** Heatmap of cell growth in a MM medium supplemented with 10 g L^−1^ glucose in the presence of different NaCl (vertical axis) and ectoine concentrations (horizontal axis), repectively, cultured in a 96-well plate for 24 h. The color shade variation in blue indicates gradient value of optical density under 600 nm (OD_600_). **b** Conceptional proof of ectoine production by *H. bluephagenesis* TD1.0 cultured in a 60MMG (60 g L^−1^ NaCl) medium used in previous studies. **c** Cloning the *ectABC* ectoine operon from *H. bluephagenesis* TD1.0 to a plasmid-based expression system pSEVA321, under the control of the *tac* promoter (P_*tac*_) inducible by IPTG. **d** Ectoine production by *E. coli* DH5α harboring an *ectABC* expression module in the presence of 100 mM sodium aspartate and three IPTG concentrations in a LB medium supplemented with 10 g L^−1^ glucose, respectively. Data are presented as mean values, error bars represent standard deviations (SD); *n* = 3 biologically independent samples. Source data underlying (**a**), (**b**), and (**d**) are provided as a Source Data file.

Previous studies reported that intracellular accumulation of ectoine plays the main role in hypersalinity resistance in most halophilic bacteria^46^, of which *H. bluephagenesis* is the one engineered as the chassis for NGIB^47^. As a metabolic target and compatible solute, cell growth characterization was performed to study the impact between ectoine and NaCl in the presence of various ectoine and NaCl concentrations, respectively (Fig. 2a). Interestingly, ectoine provides tolerance to *H. bluephagenesis* grown under high- and low-salt conditions, and minor effects were observed in a 60MMG medium regardless of ectoine concentrations (Fig. 2a and Supplementary Fig. 1).

It is important to note that the de novo synthesis pathway of ectoine has been identified, it is highly conservative across species^6^ (Fig. 1). Based on the genomic analysis of *H. bluephagenesis*, the *ectABC* gene cluster encoding the major enzymes for ectoine synthesis (Fig. 1) was annotated by several databases including RAST and KEGG. Particularly, the nucleotide blast results from NCBI database show that genes *ectA*, *ectB,* and *ectC* of *H. bluephagenesis* shared 86.46%, 86.05%, and 80.30% identity, respectively, compared with the ones from *H. elongata* DSM2581. Two strategies were employed to verify the functionality of *ectABC* cluster: (I) prototyping ectoine production by the start host, *H. bluephagenesis* TD1.0; (II) heterologous over-expressing *ectABC* for ectoine production by recombinant *E. coli*^22^.

Liquid chromatography-tandem mass spectrometry (LC-MS/MS) was applied to assay the accumulation of ectoine by *H. bluephagenesis* TD1.0 cultivated in a 60MM medium supplemented with 30 g L^−1^ glucose in shake flask studies. Results showed that the biosynthesis ectoine shared the same retention time of mass spectra with ectoine standard, which is consistent with the secondary analysis results of LC-MS/MS (Supplementary Fig. 2). Furthermore, high performance liquid chromatography (HPLC) was used to quantify the initial production of ectoine by *H. bluephagenesis* TD1.0 after cell lysis, as most halophiles accumulate ectoine intracellularly for resisting high salt circumstances (Supplementary Fig. 3). As a result, a production titer of 0.63 g L^−1^ ectoine was obtained from 9 g L^−1^ DCM containing 62 wt% PHB (Fig. 2b), confirming the function of *ectABC* cluster in *H. bluephagenesis*. Secondly, *ectABC* from *H. bluephagenesis* was cloned into the medium-copy-number plasmid pSEVA321, for heterogenous expression by recombinant *E. coli* DH5α under the control of P_*tac*_ promoter induced by IPTG (Fig. 2c). The titer of ectoine varied from 2 to 4 g L^−1^ in the presence of different IPTG concentrations for 48 h cultivation in the MME medium (Fig. 2d), further demonstrating the function of *ectABC* in *H. bluephagenesis*. Thus, more efforts would be conducted to improve the production yield of ectoine by employing various metabolic engineering approaches (Fig. 1).

### Tuning the *ectABC* expression for enhanced ectoine synthesis

*H. bluephagenesis* was generally engineered for diverse PHA productions requiring a nitrogen limited medium, or a 60MM medium supplemented with 30 g L^−1^ glucose for shake flask cultivations. As ectoine is an aspartate derived compound heavily depending on both carbon- and nitrogen-catabolism, it is necessary to reinforce the nitrogen supply via a medium modification. Even more important, transcriptional tuning of *ectABC* cluster via adding l-aspartate as precursor is significant for channeling more fluxes to ectoine, while avoiding the possibility of endogenous negative feedback control on the *ectABC* expression.

**Fig. 3:**
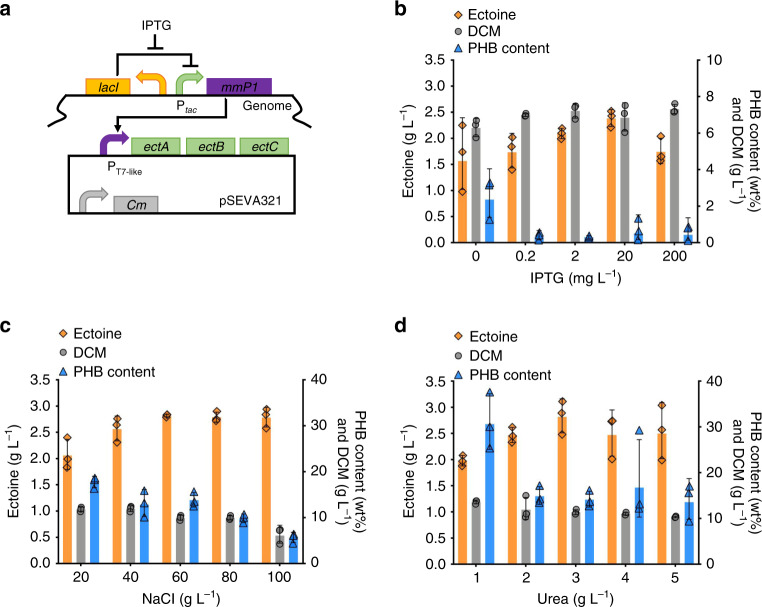
Expression tuning of *ectABC* and medium optimization for enhanced ectoine synthesis. **a** Construction of *ectABC* expression module in a plasmid-based system in *H. bluephagenesis*. A T7-like promoter (P_T7-like_) activated by MmP1 polymerase, which is controlled by P_*tac*_ promoter and induced by IPTG, namely, a T7-like inducible system, was employed to control the expression of *ectABC* operon. **b** Transcriptional tuning of *ectABC* expression unit for ectoine production by *H. bluephagenesis* TD1.0 in the presence of various IPTG concentrations, respectively, in a 60MM medium supplemented with 100 mM KCl, 100 mM sodium aspartate, 1.7 g L^−1^ citrate acid and 30 g L^−1^ glucose (short as 60MME). **c** Growth, PHB and ectoine productions by recombinant *H. bluephagenesis* TD1.0 grown in the presence of different NaCl concentrations, respectively, and induced using 20 mg L^−1^ IPTG based on the result of (**b**). **d** Effect of urea concentration as the only nitrogen source for de novo biosynthesis of ectoine and PHA production by *H. bluephagenesis* TD1.0 grown in the 60MMG medium induced by 20 mg L^−1^ IPTG. Data are presented as mean values, error bars represent standard deviations (SD); *n* = 3 biologically independent samples. Source data underlying (**b**–**d**) are provided as a Source Data file.

Based on the above understanding, a T7-like induced system was constructed to overexpress *ectABC* on a plasmid-based system in recombinant *H. bluephagenesis* TD1.0 in the presence of various concentrations of IPTG (Fig. 3a)^35^. Specifically, 100 mM aspartate was added in the cultural medium, namely MME (see methods), to ensure the metabolic fluxes flowing towards ectoine synthesis. As a result, the optimal transcriptional level for *ectABC* expression was determined after the induction of 20 mg L^−1^ IPTG, the maximum production titer of ectoine reached 2.38 g L^−1^ (Fig. 3b), indicating that a medium optimization should be conducted in the presence of 20 mg L^−1^ IPTG in shake flask studies. Therefore, factorial analysis was performed to search the optimized concentration of NaCl in the MME media for enhanced ectoine production by recombinant *H. bluephagenesis* harboring *ectABC* expression module induced by 20 mg L^−1^ IPTG. The 60MME medium containing 60 g L^−1^ NaCl demonstrated the highest 2.82 g L^−1^ ectoine when cell growth and ectoine synthesis was balanced (Fig. 3c). The ectoine titer was comparable at a higher salt concentration, but the cell growth was inhibited. In contrast, a low NaCl concentration also significant decreases the production titer of ectoine (Fig. 3c).

However, the use of aspartate as a nitrogen source is not suitable for cost-effective industrial ectoine production due to its high cost. Therefore, urea, a low-cost nitrogen source replaced aspartate for de novo synthesis of ectoine (Fig. 1). On the basis of *ectABC* flux-tuning and NaCl concentration modification, a urea optimized concentration was found to be at 3 g L^−1^, and was added to a 60MMG medium, termed 60 MMU (see methods). This resulted in a 2.83 g L^−1^ ectoine produced by recombinant *H. bluephagenesis* harboring *ectABC* expression module induced by 20 mg L^−1^ IPTG in shake flasks (Fig. 3d), which is close to that cultured in 60MME medium. Addition of urea had a negligible impact on cell growth, yet significantly decrease the PHB content when urea exceeded 1 g L^−1^. This info is an important hint for co-production of PHB and ectoine in this study.

### Deletion on degradation genes for enhanced ectoine synthesis

**Fig. 4:**
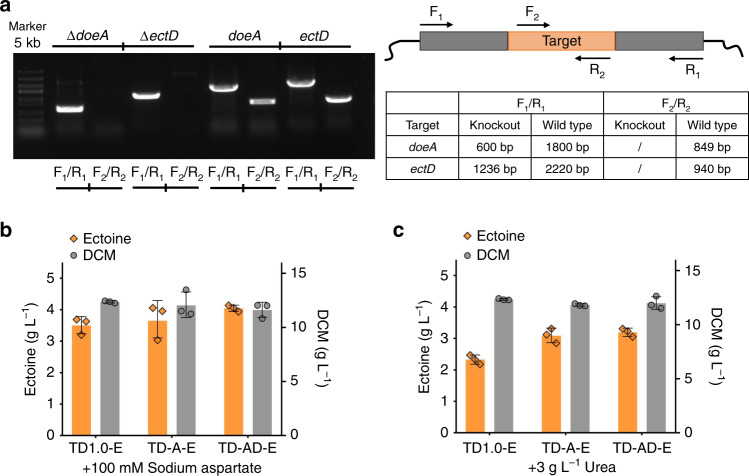
Effect of deleting ectoine degradation genes *doeA* and/or *ectD* on ectoine synthesis. **a** Primer design and colony PCR results of *doeA* and *ectD* deletion for blocking the probable degradation of ectoine. **b**, **c** Performances of ectoine accumulation by various engineered *H. bluephagenesis* TD1.0 strains, respectively, strain TD1.0: a start host, strain TD-A: single-deletion of *doeA*, and strain TD-AD: double-deletion of *doeA* and *ectD* genes. Strains harboring the e*ctABC* expression module on plasmid-based systems, namely, strain TD1.0-E, TD-A-E, and TD-AD-E, respectively, induced with 20 mg L^−1^ IPTG. **b** Cells cultured in 60MME medium, while (**c**) was grown in 60MMG medium supplemented with extra 3 g L^−1^ urea (60MMU) for de novo biosynthesis of ectoine from glucose and urea only. Data are presented as mean values, error bars represent standard deviations (SD); *n* = 3 biologically independent samples. Source data are provided as a Source Data file.

Ectoine can be degraded in two major pathways encoded by *doeA* (EC 3.5.4.44) and *ectD* (EC 1.14.11.55) (Fig. 1)^48^, forming N-α-acetyl-l-2,4-diaminobutyrate (N-α-ADABA) and 5-hydroxyectoine, respectively. Both of these two genes were found in the genome of *H. bluephagenesis* via genomic analysis, they function to reduce ectoine accumulation. It is thus necessary to eliminate these two branches for blocking the degradation of ectoine by deleting *doeA* and *ectD* (Fig. 1). A CRISPR/Cas9 mediated gene editing tool was employed to generate single-knockout *H. bluephagenesis* TD-A (Δ*doeA*), and double-knockout *H. bluephagenesis* TD-AD (Δ*doeA*, Δ*ectD*), respectively (Fig. 4a). Deletion of *doeA* and *ectD* were confirmed based on colony PCR using two primer designs (Fig. 4a). Sequences of sgRNA designed for gene deletions were listed in Supplementary Table 2, respectively.

Subsequently, ectoine production by *H. bluephagenesis* TD01 derived strains *H. bluephagenesis* TD1.0, *H. bluephagenesis* TD-A and *H. bluephagenesis* TD-AD harboring *ectABC* expression module, respectively, induced using 20 mg L^−1^ IPTG were conducted in shake flasks. Recombinant cells were cultured in 60MME (Fig. 4b) and 60MMU medium (Fig. 4c), respectively. Results showed that *doeA* deletion led to a 35% increase on ectoine titer, reaching up to 3.1 g L^−1^, and further deletion on *ectD* increased ectoine to only 3.2 g L^−1^ (Fig. 4c) when cultured in the 60MMU medium. Interestingly, only 5% and 15% increases were observed by *H. bluephagenesis* TD-A and *H. bluephagenesis* TD-AD, respectively, when cultured in the 60MME medium (Fig. 4b), probably attributed to the rich aspartate dosage. Both mutants revealed that deletions of *doeA* and *ectD* had little effect on their growth (Supplementary Fig. 4), this is helpful for scale-up studies on co-production of PHB and ectoine.

### Chromosomally transcriptional tuning of *ectABC*

**Fig. 5:**
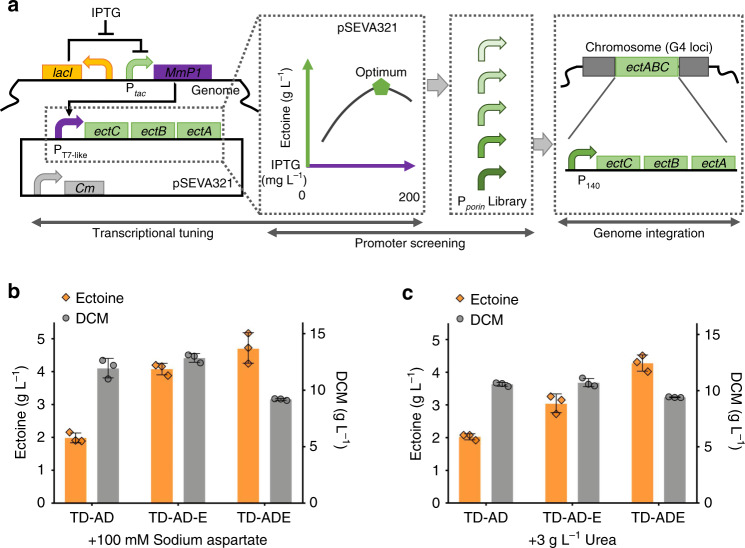
Chromosomal expression of *ectABC* in *H. bluephagenesis* TD1.0 for ectoine production. **a** Transcription-tuning design based on stimulus responsive flux-tuning approach mediated by sfGFP fluorescence intensity. Reprinted from Metabolic Engineering, 57, Jianwen Ye, Dingkai Hu, Jin Yin, Wuzhe Huang, Ruijuan Xiang, Lizhan Zhang, Xuan Wang, Jianing Han, Guo-Qiang Chen, Stimulus response-based fine-tuning of polyhydroxyalkanoate pathway in *Halomonas*, 85–95, Copyright (2020), with permission from Elsevier. **b**, **c** Shake flask results of ectoine production by recombinant *H. bluephagenesis* TD1.0 (TD-AD) and its derivates with *ectABC* expression on plasmid-based system in the presence of 20 mg L^−1^ IPTG (TD-AD-E) and chromosome-based system under the control of *porin* promoter mutant P_140_ (TD-ADE), respectively. **b** Cells cultured in a 60MME medium, while (**c**) were grown in the 60MMU medium for de novo biosynthesis of ectoine. Data are presented as mean values; error bars represent standard deviations (SD); *n* = 3 biologically independent samples. Source data underlying (**b**, **c**) are provided as a Source Data file.

**Fig. 6:**
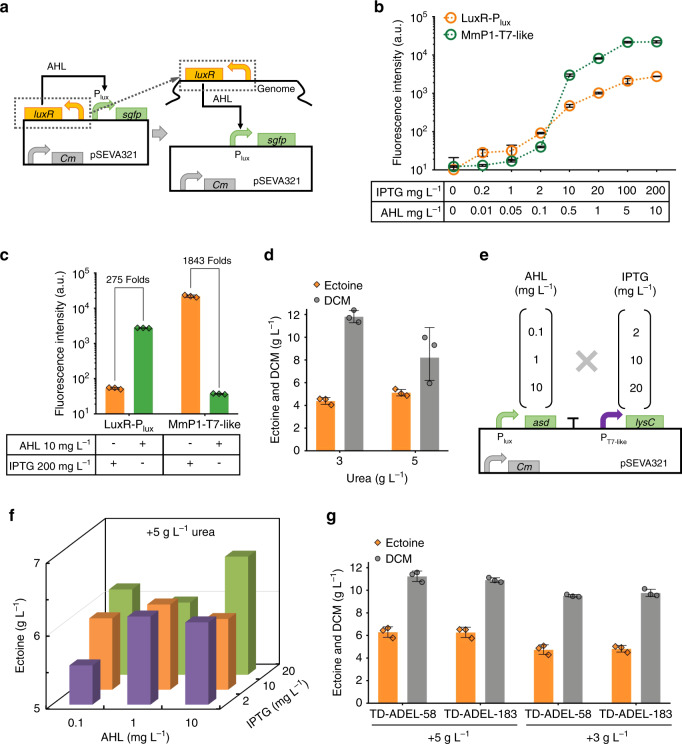
Fine-tuning of *asd* and *lysC* genes based on two orthogonal inducible systems. **a** Construction of a luxR-AHL inducible system in *H. bluephagenesis* TD1.0 by expressing regulatory protein, LuxR, on plasmid- and chromosome-based system, respectively. **b** Flow cytometer characterization of the two induced systems, luxR-AHL and T7-like, respectively. **c** Orthogonal study of luxR-AHL and T7-like inducible systems. **d** Urea on cell growth and ectoine synthesis based on *ectABC* fine-tuned strain TD-ADE. **e** Design of combinatory transcription-tuning of *asd* and *lysC* genes. **f** Transcriptional tuning of *asd* and *lysC* genes for optimal ectoine production in 500-mL shake flask cultivation in the 60MMU medium. Data are mean values of triplicates, corresponded data points with error bars were shown in Supplementary Fig. 7 independently. **g** Shake flask studies of ectoine production by engineered *H. bluephagenesis* TD1.0 strains TD-ADEL-58 and TD-ADEL-183, with fine-tuned expression modules of *asd* and *lysC* genes on chromosome based on TD-ADE in 60MMU5 and 60MMU medium, respectively. Data are presented as mean values, error bars represent standard deviations (SD); *n* = 3 biologically independent samples. Source data underlying (**b**–**d**), (**f**), and (**g**) are provided as a Source Data file.

Chromosome integration of target expression module(s) allows robust performance of cell growth under antibiotic-free cultivations. Therefore, a GFP-mediated transcriptional mapping approach developed for fine-tuning target genes in the previous study^38^ was employed to screen an appropriate constitutive promoter for chromosomal expression of the *ectABC* cluster (Fig. 5a). Specifically, fluorescent protein sfGFP serving as a global reporter for expression levels was applied to quantify and bridge the correlations cross various promoters and expression systems for efficiently screening the suitable promoter used to transcribe target expression modules, such as *ectABC*, respectively^38^ (Supplementary Fig. 5). In this study, the T7-like induced system was characterized again by expressing *sfgfp*^35^ (Fig. 6b). Simultaneously, a *porin* promoter library was constructed and characterized by expressing *sfgfp* as described in our previous study^39^.

Since the optimized expression strength of *ectABC* depends on the T7-like induced system, such as the optimized 20 mg L^−1^ IPTG induction for the T7-like system, yet IPTG is an expensive chemical not suitable for large scale applications. Thus, a constitutive *porin* promoter mutant P_140_, which exhibits a proximal expression level compared to the T7-like promoter induced by 20 mg L^−1^ IPTG in terms of the fluorescence intensity values (Fig. 6b and Supplementary Table 3), was selected to express *ectABC* module on the chromosome of *H. bluephagenesis* TD-AD (G4 loci) (Fig. 5a), forming *H. bluephagenesis* TD-ADE strain. However, the *ectABC* controlled by a stronger promoter with a fluorescence intensity value greater than 50,000 to achieve a high expression had failed to construct in *E. coli* (also for conjugation purpose). Since the expression levels of target modules on plasmid pSEVA321 are reported to be 9-fold higher than that of the chromosomal expression, which can easily lead to poor- or non-growth due to the overload of target fluxes in donor cells (*E. coli* S17-1)^38^. Ectoine production by *ectABC* fine-tuned *H. bluephagenesis* TD-ADE was carried out in shake flasks containing both 60MME and 60MMU media, forming 4.7 g L^−1^ and 4.3 g L^−1^ ectoine, respectively (Fig. 5b and c). In contrast, *H. bluephagenesis* TD1.0 harboring *ectABC* expression module under T7-like promoter encoded on a plasmid-based expression system used as positive control termed *H. bluephagenesis* TD-AD-E, produced approximately 4 g L^−1^ ectoine, significantly lower than 4.6 g L^−1^ ectoine produced by the *H. bluephagenesis* TD-ADE containing the chromosome-based *ectABC* system under the control of *porin* promoter mutant P_140_, yet much higher than the control *H. bluephagenesis* TD-AD strain forming only less than 2 g L^−1^ ectoine (Fig. 5b, c).

### Fine-tuning of *lysC* and *asd* in genome of *H. bluephagenesis*

*H. bluephagenesis* TD-ADE containing the chromosome-based and fine-tuned *ectABC* system under the control of *porin* promoter mutant P_140_, has enhanced the production of ectoine (Fig. 5). It is expected that further flux-tuning of *asd* and *lysC* genes encoding l-aspartate-semialdehyde-dehydrogenase and aspartokinase, respectively, could channel more metabolic fluxes toward ectoine synthesis (Fig. 1)^16,49^. Therefore, gene *lysC* of *Corynebacterium glutamicum* and *asd* of *H. bluephagenesis* were overexpressed in *H. bluephagenesis* TD-ADE, followed by transcription tuning to obtain better performance on ectoine accumulation.

Two inducible promoters are required including T7-like promoter to modulate the expression of *lysC* and *asd* genes, simultaneously and respectively. In this case, a quorum sensing-based regulator LuxR encoded by a *luxR* triggering the transcription of the corresponding promoter P_lux_ in the presence of acyl homoserine lactone (AHL) was constructed on a plasmid-based expression system controlled by the constitutive promoter P_J23110_^50^, together with *sfgfp* controlled by P_lux_, for the does-response functional characterization in *H. bluephagenesis* TD-ADE using flow cytometer analysis (Fig. 6a and Supplementary Fig. 6). Results exhibited 86-folds dynamic range of fluorescence intensity (FI) of sfGFP in the presence of various AHL concentrations with low basal leakiness, FI = 157 (Supplementary Fig. 6). Subsequently, the expression module of P_J23110_-*luxR* was integrated in G43 loci in *H. bluephagenesis* TD-ADE, forming *H. bluephagenesis* TD-LuxR strain (Fig. 6a). 275- and 1843-fold dynamic ranges were obtained from *H. bluephagenesis* TD-LuxR under the LuxR-AHL and T7-like induced systems, respectively, the basal leakiness of chromosome-based LuxR-AHL induced system decreased to 10 FI value (Fig. 6b). Thus, two orthogonal induced systems were successfully constructed in *H. bluephagenesis* enabling double-module modulation (Fig. 6c).

Ideally, expression of *lysC* and *asd* would be induced by IPTG and AHL, respectively, for enhanced ectoine production by consuming more urea. Before that, it is necessary to optimize urea concentration in the culture medium again. Addition of 5 g L^−1^ of urea to the 60MMG medium (namely 60MMU5) generated 17% improvement on ectoine accumulation although cell growth decreased significantly (Fig. 6d). Here, two expression modules, namely, *asd* and *lysC* controlled by P_lux_ and P_T7-like_, respectively, were constructed on a plasmid-based system for transcriptional tuning (Fig. 6e). The induction concentrations of IPTG and AHL did have a significant effect on ectoine production. For example, an induction using 20 mg L^−1^ IPTG and 10 mg L^−1^ AHL generated the highest 6.6 g L^−1^ ectoine, 29% higher compared to that produced by *H. bluephagenesis* TD-ADE (Fig. 6d, f, Supplementary Fig. 7). Using the GFP-mediated transcription-tuning approach, *porin* promoter mutants P_226_ and P_140_ were selected to control the expression of *lysC*, and P_58_ and P_183_ for *asd* on the chromosome of *H. bluephagenesis* TD-ADE.

However, the expression module encoding *lysC* controlled by P_140_ failed to construct in the donor *E. coli* as also mentioned previously. Yet, two strains, *H. bluephagenesis* TD-ADEL-58 and TD-ADEL-183, were successfully grown for shake flask studies using the 60MMU5 medium (Fig. 6g). The two strains produced almost 6.3 g L^−1^ ectoine after 48 h cultivation in the 60MMU5 medium, over 30% higher compared to that cultured in the 60MMU medium. Due to the failure of P_140_-*lysC* construction, a weaker transcription strength of *lysC* controlled by P_226_ probably weakens the flux for l-β-aspartyl phosphate synthesis, which coupled with *asd* controlled by P_183_, it cannot bring significant increase on ectoine when grown in both 60MMU and 60MMU5 media, respectively. Thus, *H. bluephagenesis* TD-ADEL-58 became a better option for fed-batch studies in 7 L lab-scale bioreactor. Similarly, considering the fed-batch study for co-production of PHB and ectoine, *H. bluephagenesis* TD-ADE with genomic expression of *lysC* and *asd* genes were performed in a 96-well plate to investigate the effect on growth scale-up (Supplementary Fig. 4).

### Fed-batch study for co-production of PHB and ectoine

**Fig. 7:**
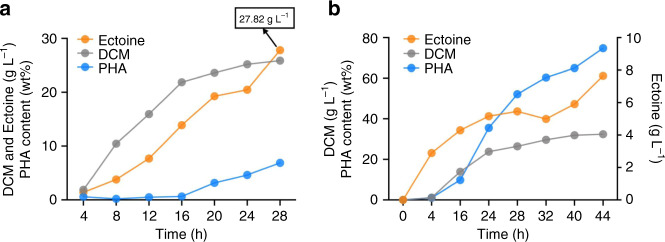
Ectoine production by chromosomally engineered *H. bluephagenesis* TD-ADEL-58. **a** Growth, PHB and ectoine production by chromosomally engineered *H. bluephagenesis* TD1.0 strain TD-ADEL-58 fine-tuned with the ectoine biosynthesis pathway, including three individual expression units controlled by *porin* promoters of various strengths, respectively. **b** Co-production of ectoine and PHB by chromosomally engineered *H. bluephagenesis* TD1.0 (TD-ADEL-58). Nitrogen source in feeding solution Feed-I/II was reduced to enhance PHB accumulation compared to (**a**) with poor PHB accumulation. Data are generated from one fed-batch fermentation in a 7-L bioreactor under open unsterile conditions without means and error bars. Source data are provided as a Source Data file.

*H. bluephagenesis* TD-ADEL-58 was grown for 28 h for ectoine production under open unsterile conditions in a 7 L bioreactor. 28 g L^−1^ ectoine were produced from this fed-batch study, which is over 4-folds higher than that of the shake flask results (Fig. 7a). The yield of ectoine on glucose and volumetric productivity reached approximately 0.21 g g^−1^ and 1.0 g L^−1^ h^−1^, respectively. Ectoine accumulation tendency was highly consistent with cell growth in terms of DCM, indicating the growth-coupled property of ectoine biosynthesis. Meanwhile, PHB accumulation was maintained at a low level during the first 16 h of cultivation before its increase afterward (Fig. 7a). In contrast, a fed-batch fermentation was executed for 28 h using *H. bluephagenesis* TD-ADE to verify the effect of fine-tuning *asd* and *lysC* gene on ectoine production, leading to 17 g L^−1^ ectoine, which was over 30% lower than results of *H. bluephagenesis* TD-ADEL-58 (Supplementary Fig. 8).

To achieve co-production of ectoine and PHB by *H. bluephagenesis* TD-ADEL-58, a tailor-made feeding solution was designed to enhance PHB accumulation during the stationary phase using reduced nitrogen dosage in the second phase feeding solution (Feed-II). Similarly, a fed-batch fermentation was carried out in the 7 L bioreactor to co-produce ectoine and PHB in different growth phases. After a 44 h cultivation, the DCM reached 32 g L^−1^ containing 75% PHB, together with 8 g L^−1^ ectoine (Fig. 7b). It was interesting to observe that ectoine biosynthesis maintained active in the stationary phase during the fed-batch cultivation (Fig. 7b). It is expected that more bioprocess optimizations should lead to even higher ectoine and PHB production under balancing nitrogen supply in different growth phases^51,52^.

## Discussion

Next generation industrial biotechnology (NGIB) based on halophiles provides low-cost and convenient processing conditions for production of chemicals^47^. The most successful case has been exemplified by 5000 L scale fermentor for PHA production^27^. It has been interesting to look for other examples to test possibilities of NGIB for production of non-polymer products, or even high-value added example. Ectoine, a high-value added product derived from l-aspartate synthesized by some microorganisms, has attracted increasing attentions due to its outstanding functions of stabilizing proteins and nucleic acids^53^. To date, many ectoine commercial applications, such as cosmetic ingredient, have been developed with high-value. However, ectoine production by wild type and engineered microorganisms yields very low productivity. Some metabolic engineering approaches have been adopted for enhanced productions of amino acids-related compounds, including ectoine^54^, l-threonine and l-lysine^37^, including new tools for strain engineering, such as dynamic and static optimization for flux enforcement^55^: stimulus response-based transcriptional controls^56,57^ and promoter and RBS optimizations et al^40^. *H. bluephagenesis*, a high-salt tolerant halophile able to grow to a high cell density with scalability, is a promising host for ectoine synthesis because of its being a natural ectoine producer harboring the *ectABC* cluster in its genome. Recently, many efforts have been made to establish various genetic manipulation tools and flux-tuning approaches for improving and diversifying this NGIB chassis^35,38^. This study has demonstrated successful and high yield on ectoine production by chromosomally engineered *H. bluephagenesis* utilizing static optimization strategies, reaching close to 30 g L^−1^ ectoine from a fed-batch fermentation process (Fig. 7a).

Ectoine biosynthesis pathway of *H. bluephagenesis* was clearly identified by heterologous expression in recombinant *E. coli*, in which strong enzymatic activities of *ectABC* cluster were found compared with previous studies^14,22^ (Figs. 1, 3, and 4). Cell growth studies uncovered the enhanced resistance of cells under both low- and high-salt concentrations in the presence of ectoine, respectively. Furthermore, competitive ectoine degradation pathways were removed by eliminating the related genes *doeA* and *ectD*^48^, resulted in 89% improvement on ectoine production from shake flask cultivations. Two pathway branches reported by previous studies involved in l-threonine and l-lysine synthesis were retained in consideration for the benefits of ectoine co-production with PHB^43,51^, a growth-dependent intracellular product, as the disruption of l-threonine and l-lysine will weaken cell growth and thus PHB accumulation.

We thus attempted to utilize transcriptional balancing strategies to boost fluxes towards ectoine synthesis. In contrast to translational regulation, transcriptional regulation could be more efficient to be carried out with minor constructs during strain engineering by one-step fine-tuning of target expression modules based on stimulus response-based flux-tuning design^38^. Five genes involved in ectoine synthesis pathway were grouped into three individual expression units, including *ectABC, lysC and asd*, for transcriptional fine-tuning (Figs. 1, 4). It has to be noted that quorum-sensing-based inducible system, LuxR-AHL, was firstly constructed and employed to achieve combinatory transcriptional tuning together with T7-like system in *H. bluephagenesis* (Figs. 5, 6). Specifically, expressions of two simple constructs, p321-P_J23110_-*lacI*-P_T7-like_-*ectABC* and p321-P_T7-like_-*lysC*-P_lux_-*asd*, could simulate countless transcriptional combinations of target modules replacing thousands of variants construction. Finally, 9.8-folds improvement of production titer of ectoine was achieved in a shake flask study by recombinant *H. bluephagenesis* harboring fine-tuned synthesis pathway of ectoine in genome compared with 0.64 g L^−1^ titer yielded by the start host (Fig. 6). Subsequently, an ectoine titer of approximately 30 g L^−1^ was achieved under open unsterile fed-batch fermentation conducted in the 7-L bioreactor.

Co-production of PHB and ectoine allows harvests of intracellular bioplastic PHB and extracellular small molecular chemical ectoine, it is thus economically interesting. However, it is not sure if the two products competing for metabolic fluxes could reduce their yields. A conceptional proven study was conducted in a lab-scale fermentation. Both ectoine and PHB are growth-associated products derived from aspartate and acetyl-CoA when nitrogen source supply is abundant, approximately 28 g L^−1^ ectoine and 25 g L^−1^ DCM together with 6% PHB were obtained, respectively, during a 28 h of growth (Fig. 7a). In contrast, under a reduced nitrogen source, PHB accumulation was increased to 75% of the DCM in the cells producing also 8 g L^−1^ ectoine (Fig. 7b). In both cases, the recombinant *H. bluephagenesis* was grown to over 30 g L^−1^ DCM that are not the highest reported by this organism^27,58^ (Fig. 7). Further efforts to increase the recombinants’ growth density should lead to much improved ectoine and PHB productions.

Etoine as a representative product of l-aspartate compound family with a high value, should be able to produce at a competitive cost after fine-tuning of every single gene of *ectABC* cluster employing SR-FT approach by developing more suitable inducible systems^59^. Although static optimization is a widely used strategy for enhanced production of metabolic targets, recent progresses focused on bifunctional dynamic control provide a strong hint for improving the production of ectoine. Since co-production of PHB and ectoine seems to be competing for flux, the efficiency to activate target pathways dynamically for PHB and ectoine must be considered during the fermentation processes^60,61^. In addition, the bioprocessing optimization, such as feeding solution optimization^62^ and cascaded bioprocessing^52^, is a promising strategy to obtain simultaneously enhanced production yields for ectoine and PHB. Additionally, attempts of enhancing export system^48^ and developing amino acids responsive regulators^57^ could also be applied to achieve further improvement on ectoine and PHB production under cost-effective bio-manufactural process based on the NGIB chassis.

In conclusion, the halophile NGIB chassis can be effectively used for effective production of ectoine. Engineering the ectoine pathways improved ectoine production effectively. Co-production of intracellular PHA and non-polymer chemicals is possible using the halophilic chassis.

## Methods

### Strains culture and chemicals

LB medium is composed of (g L^−1^): 10 trypton, 5 yeast extract and 10 NaCl. 60LB medium is derived from LB medium supplemented with 60 g L^−1^ NaCl. 60MMG medium consists of (g L^−1^): glucose 30, NaCl 60, yeast extract 1, (NH_4_)_2_SO_4_ 0.25, MgSO_4_ 0.2, Na_2_HPO_4_·12H_2_O 9.65, KH_2_PO_4_ 1.5, trace element solution I 10 mL L^−1^ and trace element solution II 1 mL L^−1^. The composition of trace element solution I consists of (g L^−1^): Fe(III)-NH_4_-citrate 5, CaCl_2_ 2, HCl 1 M. The trace element solution II (mg L^−1^) consists of: ZnSO_4_·7H_2_O 100, MnCl_2_·4H_2_O 30, H_3_BO_3_ 300, CoCl_2_·6H_2_O 200, CuSO_4_·5H_2_O 10, NiCl_2_·6H_2_O 20, and NaMoO_4_·2H_2_O 30. 60MME is derived from 60MMG medium supplemented with 100 mM sodium aspartate with additional 100 mM KCl, and 1.7 g L^−1^ citric acid. Alternatively, 100 mM sodium aspartate in 60MME medium was replaced by 3 and 5 g L^−1^, respectively, to form 60MMU and 60MMU5 media for de novo synthesis of ectoine for shake flask studies. Ectoine of analytical purity purchased from Sigma-Aldrich (St. Louis, MO, USA) was used as standard for HPLC analysis for fermentative ectoine quantitation.

*H. bluephagenesis* TD1.0 derived from *H. bluephagenesis* TD01, isolated from Aydingol Lake of China, was used to engineer for production of ectoine in this study. *Escherichia coli* S17-1pir, the donor strain for conjugation, was cultivated in LB medium. Seed preparation of *H. bluephagenesis* and its derivates in this study were cultured in 60LB medium. Co-production of ectoine and PHA in shake flasks by recombinant *H. bluephagenesis* TD were cultivated in 60MMG, 60MME, 60MMU and 60MMU5 media, respectively.

All the derivates from *H. bluephagenesis* TD1.0, wild type *H. bluephagenesis* TD integrated with expression module of MmP1 RNA polymerase induced by IPTG, were listed in Supplementary Table 1. Firstly, *doeA* and *ectD* gene encoding bypass (ectoine degradation) enzymes were deleted, forming *H. bluephagenesis* TD-AD strain. Then, expression modules including *ectABC*, *lysC* and *asd* cluster were integrated into the chromosome of *H. bluephagenesis* one-by-one at G4, G7 and G43 locus, respectively, forming *H. bluephagenesis* strains TD-ADE, TD-ADEL-58, and TD-ADEL-183.

### Plasmids construction

Plasmids were constructed to carry functional gene expressions encoded in vector pSEVA321^63^. All the constructs were assembled by Gibson Assembly kit, and conjugated into *H. bluephagenesis* derivates. DNA extraction was carried out by using Tiangen Plasmid Extraction kit (Shanghai, China) and Omega Gel Purification kit (USA). DNA oligonucleotides were synthesized by Zixi Biotechnology (Beijing, China). Genetic design and sequence reading were performed by Snap Gene (v2.3.2).

All the plasmids were constructed by Gibson Assembly kit. For gel purification, the Omega gel purification kit was used, and the Tiangen plasmid extraction kit was used to obtain the plasmids. All the plasmids used in this study and genes required fine-tuning to obtain enhanced ectoine production were listed in Supplementary Table 1. The sequences of sgRNA used for genome editing in the study were listed in Supplementary Table 2. CRSPR-based genomic editing approach was employed to construct various recombinant strains by gene knockout and gene integration^34^. Firstly, plasmid pQ08 containing Cas9 expression module was transformed into recombinant *H. bluephagenesis* by conjugation. Followed by the conjugation of constructs derived from pSEVA241 vector carrying gRNA and homologous arms (500 bp), positive colonies were screened by PCR test and confirmed by DNA sequencing after the discard of plasmids by passaging the cell cultures in 60 LB medium without antibiotics for 3–5 generations.

### Conjugation

Firstly, plasmids were constructed by *E. coli* DH5α, subsequently transformed into *E. coli* S17-1, the donor strain for conjugation. Then, donor cells harboring target constructs were cultivated for 12–14 h in 150-mL shake flasks containing 20 mL LB medium, and recipient cells, recombinant *H. bluephagenesis*, were cultivated in 60LB medium overnight. Both of the donor and recipient cells were centrifugated and harvested under 4 ^o^C, washed with a fresh medium and recovering to the same volume that sampled. Subsequently, cells were mixed (1:1 volume) and dropped on antibiotic-free 20-LB agar plates, followed by 6–8 h incubation at 37 ^o^C for conjugation. Finally, conjugated cells from the lawn were suspended with 60LB medium and spread on 60LB agar plates containing relevant antibiotics for 36 h incubation to obtain positive colonies.

### Characterization of LuxR-AHL and T7-like inducible systems

LuxR-AHL inducible system was first constructed on plasmid-based expression system in *H. bluephagenesis* TD1.0 harboring plasmid p321-P_J23110_-*luxR*-P_lux_-*sfgfp* to characterize the performance in the presence of various AHL (OC6, from Sigma) concentrations, respectively. Then, *luxR* expression module was integrated into the chromosome of *H. bluephagenesis* TD-ADE at G43 loci, forming TD-LuxR strain, followed by the characterization of LuxR-AHL and T7-like induced systems in *H. bluephagenesis* TD-LuxR strain harboring plasmids, p321-P_lux_-*sfgfp* and p321-P_T7-like_-*sfgfp* after conjugation. Cells carrying target expression vessels were plated on 60LB agar plate added with relevant antibiotics for 16–20 h of incubation at 37 °C. Subsequently, single colony was inoculated into 1 mL 60LB medium for 12 h cultivation in 96-deep-well plate covered with sealing films (BF-400-S; Thermo-Shaker, Aosheng, 37 °C, 1000 rpm). Then, 5 µL cell culture was inoculated into a new plate supplemented with appropriate antibiotics in presence of a spectrum of inducer. After 12 h incubation, cell cultures were diluted 250-folds using phosphate-buffered saline solution (PBS) for flow cytometry analysis.

Positive fluorescent cells were recorded by flow cytometer (LSRFortessa4, BD bioscience, USA) at the rate of 0.5 µL s^−1^ for 20 s with at least 30,000 cell captured in total of each sample, and the start host, *H. bluephagenesis* TD1.0, was used as the negative control groups to modify the fluorescence levels of experimental groups. Intracellular sfGFP protein was excited under 488 nm, and cells were captured on the signal channels of FITC, FSC (forward scatter) and SSC (side scatter). The FlowJo (v7.6) software was used to process the raw data for obtaining mean value of fluorescence intensity and percentage of positive cells.

### Cell growth study

To study the effects of ectoine and NaCl on cell growth, single colony of recombinant *H. bluephagenesis* TD1.0 was first picked as an inoculum for a 12 h incubation in 1 mL 60LB medium in a 96-deep-well plate (Corning/USA, 1 mL 60LB medium) at 37 °C. Subsequently, seed cultures were diluted 200-folds with fresh MM medium supplemented with 20 g L^−1^ in the presence of various ectoine and NaCl concentrations in a new 96-well plate (flat bottom, 250 µL medium per well) for online recording of optical density at 600 nm (OD_600_) for 48 h.

To characterize cell growth of recombinant *H. bluephagenesis* in this study, chromosomally engineered strains were pre-cultured in a 60-LB medium in the 96-deep-well plate for 12 h by picking a single colony as an inoculum. Then, seed cultures were diluted 200-folds with a fresh 60LB medium in a new 96-well plate (flat bottom, 250 µL medium per well) for online monitoring of optical density at 600 nm (OD_600_) for 24 h. The cell growth characterization was carried out using a micro-plate reader (Varioskan Flash, Thermo Scientific).

### Shake flask and fed-batch studies

For shake flask studies, recombinant *H. bluephagenesis* harboring target expression modules on plasmid- or chromosome-based systems were pre-cultured overnight to obtain seed cultures (OD_600_ reached 2.5 ± 0.2) from a single colony, respectively. Subsequently, 2.5 mL seed culture were inoculated into 50 mL 60MM medium supplemented with 30 g L^−1^ glucose and nitrogen source (urea or sodium aspartate) whenever necessary. 25 µg mL^−1^ chloramphenicol was added to the medium grown with *H. bluephagenesis* harboring the plasmid-carried expression systems. Cell cultures were harvested for dry cell mass (DCM), ectoine and PHB content assays after 48 h cultivation. Additionally, functional verification of gene cluster *ectABC* from *H. bluephagenesis* was performed using the same procedure of recombinant *H. bluephagenesis* grown in LB medium and recombinant *E. coli* DH5α harboring plasmid p321-J_23110_-*lacI*-P*tac*-*ectABC*.

For fed-batch studies, cells were grown overnight in 60LB medium in 500 mL shake flasks to obtain 300 mL seed cultures (OD_600_ reached 2.5 ± 0.2) as inoculums in a fed-batch study conducted in a 7-L bioreactor (NBS Bioflo3000, New Brunswick, USA). A 3 L 60 MM medium supplemented with 3 g L^−1^ CO(NH_2_)_2_, 10 g L^−1^ yeast extract and 20 g L^−1^ glucose was used as an initial medium for fermentation. During the fermentation process, dissolved oxygen (DO%) was maintained at ~30% of air saturation by injecting air with a maximum flow rate of 1 VVM (air volume per culture volume per min) by coupling the agitation of less than 800 rpm. pH was adjusted using 5 M NaOH solution. Feed solution of the first phase (Feed-I) is composed of 200 g glucose, 8 g yeast extract and 32 g urea (24 g of urea for co-production purpose). For ectoine production, the second phase feeding solution (Feed-II) consists of 200 g glucose, 4 g yeast extract and 28 g urea, while fed nutrients composed of 200 g glucose, 4 g yeast extract and 8 g urea for co-production of ectoine and PHB. And Feed-III containing only 800 g L^−1^ glucose was fed when Feed-II ran out during the fermentation for co-production of ectoine and PHB. Feed solutions were fed to maintain the residual glucose at 6–10 g L^−1^ (measured by SANNUO medical glucometer, China). All batches of 7-L fermentation were conducted under non-sterilized open condition at 37 °C. Then, cells sampled of every time point were harvested for cell mass, ectoine and PHB assays.

### Assays of dry cell mass and PHB content

First, cells were harvested from 30 mL fermentative broth during a fed-batch study or at the end of the shake flask study via centrifugation at 12,200*g* (CR 21GIII, HITACHI, Japan) for 15 min, subsequently suspended and washed twice with distilled water. A dry cell mass was calculated by measuring the mass of harvested cells after 24 h of lyophilization under −60 °C and 100 Pa. For PHB content assays, 30–40 mg lyophilized cells in powder form were sampled for methanolysis under 100 °C for 3.5 h supplemented with 2 mL chloroform and 2 mL methanolysis solution (85 wt% methanol, 15 wt% H_2_SO_4_, and 1 g L^−1^ benzoic acid) mixture^48^. Then, 1 mL water was added to the methanolysis solution after cooling down to room temperature (25 °C) for extraction and phase separation. 1 mL of the heavy (low) phase was sampled for PHB content calculation using GC (gas chromatography, GC-2014, SHIMADZU, Japan) analysis. 15 and 20 mg pure PHB (3HB standard from Sigma-Aldrich) were used as standards.

### Ectoine assays

Five millimeters of fermentative broth from 30 mL cell cultures during fed-batch study or at the end of shake flasks were diluted 10-folds (50-folds for the fed-batch study) in a 50 mL tube. Subsequently, cells were disrupted by a high pressure homogenizer and centrifugated at 12,200*g* for 10 min. Then, the supernatants were filtrated once by 0.22 μm filter membrane for ectoine quantification using high-performance liquid chromatography (HPLC, SHIMADZU, Japan) equipped with a C18 column based on acetonitrile/water mixture (70:30, v/v) at a flow rate of 1 mL per min as the mobile phase. UV (ultraviolet) detector (210 nm) was used for ectoine detection. The HPLC-MS analysis was carried out using OrbiTrap Q-Exactive LC-MS (Thermo Scientific) under the same conditions.

### Reporting summary

Further information on research design is available in the Nature Research Reporting Summary linked to this article.

## Supplementary information

Supplementary Information

Peer Review File

Reporting Summary

Description of Additional Supplementary Files

Supplementary Data 1

Supplementary Data 2

Supplementary Data 3

Supplementary Data 4

Supplementary Data 5

Supplementary Data 6

Supplementary Data 7

Supplementary Data 8

Supplementary Data 9

Supplementary Data 10

Supplementary Data 11

Supplementary Data 12

Supplementary Data 13

## Data Availability

The datasets generated and analyzed during the current study are available from the corresponding authors upon request. A reporting summary for this Article is available as a Supplementary Information file. The datasets generated and analyzed during the current study are available from the corresponding author upon request. Plasmids sequences are available in Supplementary Data 1–13. Source data are provided with this paper.

## Source data

Source Data
