# Supplementary material for: Rational flux-tuning of Halomonas bluephagenesis for co-production of bioplastic PHB and ectoine
Source: Nat Commun. 2020 Jul 3;11:3313. doi: 10.1038/s41467-020-17223-3 (PMC7334215; doi:10.1038/s41467-020-17223-3)
Supplement: Supplementary file 1 — Supplementary Information [file 41467_2020_17223_MOESM1_ESM.pdf]

Rational flux-tuning of *Halomonas bluephagenesis* for co-production of bioplastic PHB and ectoine

Ma *et al.*

Supplementary Table 1. Strains, plasmids and genes used in this study.

Strains/Plasmids/ Genes	Description	Reference/Source
**Strains**		
E. coli S17-1 pir	A vector donor used for conjugation, harbors the *tra* genes from plasmid RP4 in the chromosome	Simon *et al.*[1]
H. bluephagenesis TD01	*H. bluephagenesis* TD01, wild type halophile, isolated from a salt lake in China	Tan *et al.*[2]
H. bluephagenesis TD1.0	*H. bluephagenesis* TD01 with the integration of RNA polymerase expression module, P_{J23110}-*lacI*-P_{tac}-*MmP1*, to activate the transcription of T7-like promoter in presence of IPTG.	Zhao *et al.*[3]
TD1.0-E	*H. bluephagenesis* TD1.0 strain harboring plasmid p321-$P_{T7\text{-like}}$-*ectABC*	This study
TD-A	*H. bluephagenesis* TD1.0 derivative with *doeA* deleted	This study
TD-A-E	TD-A harboring plasmid p321-$P_{T7\text{-like}}$-*ectABC*	This study
TD-AD	TD-A derivative with *ectD* deleted	This study
TD-AD-E	TD-AD strain harboring plasmid p321-$P_{T7\text{-like}}$-*ectABC*	This study
TD-ADE	TD-AD integrated 'P_{140}-*ectABC*' part on G4 site	This study
TD-ADEL-58	TD-ADE integrated 'P_{226}-*lysC*' part on G7 site and 'P_{58}-*asd*' part on G43 site	This study
TD-ADEL-183	TD-ADE integrated 'P_{226}-*lysC*' part on G7 site and 'P_{183}-*asd*' part on G43 site	This study
TD-LuxR	TD-ADE integrated 'P_{J23110}-*luxR*' part on G43 site	This study
**Plasmids**		
pSEVA321	p321, RK2 replication origin, containing the oriT sequence for conjugate transformation, an expression vector in *H. bluephagenesis* TD01 strain and its derivates, Cm^R. The lowest copy number plasmid used in this study.	Silva-Rocha *et al.*[4]
pSEVA241	pRO1600 / ColE1 replicon, containing the oriT sequence for conjugate transformation, an expression vector in *H. bluephagenesis* TD01 strain and its derivates, Km^R and Sp^R. The highest copy number plasmid used in this study.	Qin *et al.*[5]
pQ08	pSEVA321 derivative, *S. pyogenes cas9*, Cm^R	Qin *et al.*[5]
pQ41	pSEVA241 derivative, P_{J23119}-sgRNA (IR 00227-00228-G4), 500 bp donor, Km^R and Sp^R	Qin *et al.*[5]
pQ44	pSEVA241 derivative, P_{J23119}-sgRNA (IR 02654-02655-G7), 500 bp donor, Km^R and Sp^R	Qin *et al.*[5]

pQ133	pSEVA241 derivative, P_{J23119}-sgRNA (IR 00653-00654-G43), 500 bp donor, Km^R and Sp^R	Qin *et al.*[5]
p321-P_{tac}-*ectABC*	pSEVA321 derivative, *ectABC* driven by P_{tac}, Cm^R	This study[a]
p321-P_{J23110}-*luxR*-P_{lux}-*sfgfp*	pSEVA321 derivative, *luxR* driven by P_{J23110} and *sfgfp* driven by P_{lux}, Cm^R, namely LuxR-AHL inducible system.	This study[a]
p321-P_{lux}-*sfgfp*	pSEVA321 derivative, *sfgfp* driven by P_{lux}, Cm^R, namely LuxR-AHL inducible system.	This study[a]
p321-P_{J23110}-*lacI*-$P_{T7\text{-}like}$-*sfgfp*	pSEVA321 derivative, *lacI* driven by P_{J23110} and *sfgfp* driven by $P_{T7\text{-}like}$, Cm^R, namely LuxR-AHL inducible system.	This study[a]
p321-P_{J23110}-*lacI*-$P_{T7\text{-}like}$-*ectABC*	pSEVA321 derivative, *lacI* driven by P_{J23110} and *sfgfp* driven by $P_{T7\text{-}like}$, namely T7-like inducible system, Cm^R	This study[a]
p321-$P_{T7\text{-}like}$-*lysC*-P_{lux}-*asd*	pSEVA321 derivative, *lysC* driven by $P_{T7\text{-}like}$ and *asd* driven by P_{lux}, Cm^R	This study[a]
pQ133-LuxR	pSEVA241 derivative, P_{J23119}-sgRNA (IR 00653-00654-G43)::P_{J23110}-*luxR*, 500 bp donor, Km^R and Sp^R	This study[a]
pQ133-A	pSEVA241 derivative, P_{J23119}-sgRNA (*doeA*-QG10), 500 bp donor, Km^R and Sp^R	This study[a]
pQ133-D	pSEVA241 derivative, P_{J23119}-sgRNA (*ectD*-QG13), 500 bp donor, Km^R and Sp^R	This study[a]
pQ41-ABC	pSEVA241 derivative, P_{J23119}-sgRNA (IR 00227-00228-G4)::P_{140}-*ectABC*, 1 kp donor, Km^R and Sp^R	This study[a]
pQ44-LysC	pSEVA241 derivative, P_{J23119}-sgRNA (IR 02654-02655-G7)::P_{226}-*lysC*, 500 bp donor, Km^R and Sp^R	This study[a]
pQ133-Asd-58	pSEVA241 derivative, P_{J23119}-sgRNA (IR 00653-00654-G43)::P_{58}-*asd*, 500 bp donor, Km^R and Sp^R	This study[a]
pQ133-Asd-183	pSEVA241 derivative, P_{J23119}-sgRNA (IR 00653-00654-G43)::P_{183}-*asd*, 500 bp donor, Km^R and Sp^R	This study[a]
**Genes**		
luxR	LuxR family transcriptional regulator gene of *Vibrio fischeri* ES114 (AAW87995.1)[b]	Zeng *et al.*[6]
ectA	L-2,4-diaminobutyrate acetyltransferase of *H. bluephagenesis* TD1.0 (EGP18461.1)[b]	This study
ectB	L-2,4-diaminobutyrate transaminase of *H. bluephagenesis* TD1.0 (EGP18460.1)[b]	This study
ectC	Ectoine synthase of *H. bluephagenesis* TD1.0 (EGP18459.1)[b]	This study

doeA	Ectoine hydrolase of *H. bluephagenesis* TD1.0 (EGP21610.1) [b]	This study
ectD	Ectoine hydroxylase of *H. bluephagenesis* TD1.0 (EGP18127.1) [b]	This study
lysC	Aspartokinase of *Corynebacterium glutamicum* (BAB97644.1) [b]	This study
asd	L-aspartate-semialdehyde-dehydrogenase of *H. bluephagenesis* TD1.0 (EGP19445.1) [b]	This study

[a] Plasmids are provided in a Supplementary Data 1-13.
[b] Gene IDs from Genebank.

Supplementary Table 2. sgRNA sequences used in this study.

Purposes	Target site	Guide sequence	PAM
doeA deletion	*doeA*	GCGAAATGGCGTATCGTCCC	AGG
ectD deletion	*ectD*	TTAACCACCGCATCACGACG	CGG
ectABC insertion	G4 loci	TTCACCTAGCTAGATGAGAC	AGG
lysC insertion	G7 loci	ACACCATTACGGGGGTGTCA	CGG
asd/luxR insertion	G43 loci	GTGTCCATGTTTTTGACGCG	TGG

Supplementary Table 3. Promoter strengths of *porin* promoter mutants constructed on different genomic sites in *H. bluephagenesis* TD01.

Promoter No.	Mean value of FI[a] on plasmid pSEVA321	Mean value of FI on genomic locus G4/G7/G43[b]	Mutated sequence[c]
140	50933	5659/6367/5659	GTATA**AGA**
226	31600	3511/3950/3511	GTATA**A**AG
183	20333	2259/2542/2259	GTATAG**CA**
58	12810	1423/1601/1423	**A**TATAGAG

[a] FI: fluorescence intensity of sfGFP;

[b] Mean value of FI of *sfgfp* expressed on plasmid pSEVA321 divided by 9 (G4), 8 (G7) and 9 (G43), respectively. Correlations of expression levels between plasmid-based and chromosome-based system of G4 and G7 locus were characterized by Ye *et al.*[7]. , and G43 loci displayed similar expression strength with G4 reported by Qin *et al.*[5].

[c] Mutated sequences (letter in bold with gray background) of the core region of *porin* promoter in previous study[8].

Supplementary Table 4. Primers used in this study.

Name (-F/-R) [a]	Sequence (5’-3’)	Notes [b]
EctABC-F	gagaaagaggagaaatactagatgagtacgccaataacaccttttacccc	Construction of p321-P$_{tac}$-*ectABC* and p321-P$_{J23110}$-*lacI*-P$_{T7\text{-}like}$-*ectABC*
EctABC-R	tttgatgcctggcttatcattactcacccgcgggtgctg	
p321-Ptac-F	agcacccgcgggtgagtaatgataagccaggcatcaaataaaa	
p321-Ptac-R	catctagtatttctcctctttctctagtattaa	
p321-Bsfgfp-F	attcaccaccctgaattgactctc	Construction of p321-P$_{J23110}$-*luxR-sfgfp*
p321-Bsfgfp-R	gaattctaattggtaacgaatcagacaattgacg	
LuxR-F	tctgattcgttaccaattagaattcgagagcgttcaccgacaaac	
LuxR-R	agagagtcaattcagggtggtgaatatgaaaaacataaatgccgacgacacatac	
p321-Plux-sfgfp-F	gaaaatggtttgttactttcgaataaaagctgtcaccggatgtgc	Construction of p321-P$_{lux}$-*sfgfp*
p321-Plux-sfgfp-R	ttgcgtaaacctgtacgatcctacaggtcagccaacgatcgttccgat	
p321-PT7-like-F	ttcgtcaggccacatagctttcttgttc	Construction of p321-P$_{J23110}$-*lacI*-P$_{T7\text{-}like}$-*sfgfp*
p321-PT7-like-R	gaattcgcgcggccgcggccta	
LacI-F	ggccgcggccgcgcgaattctcactgcccgctttccagtcgggaaacct	
LacI-R	aagctatgtggcctgacgaagcggcgcgccatcgaatggcgcaa	
p321-F	catctgttttcttgcaagattactagtagcggccgctgcag	Construction of p321-P$_{T7\text{-}like}$-*lysC*-P$_{lux}$-*asd*
p321-R	tgataagccaggcatcaaataaaacgaaag	
lysC-F	atttgatgcctggcttatcattagcgtccggtgcctgcat	
lysC-R	gagagcgttcaccgacaaacaacagataaa	
asd-F	gtttgtcggtgaacgctctcgccacatagctttcttgttctgat	
asd-R	atcttgcaagaaaacagatggcaagc	
G43-backbone-F	aattctaattggtaacgaatcagacattatcttatacagtataactattgtacataaatcagcgtacat	Construction of pQ133-LuxR
G43-backbone-R	tttgcgccattcgatggcgcgccgctaagtgtcgggcagcgagataa	
LuxR-F	gcggcgcgccatcga	
LuxR-R	gtctgattcgttaccaattagaattcgagagcgttcacc	
Donor-F	agccgtcgtgactgggaaaa	Construction of pQ133-A
Donor-R	ccgggttcaaaaaaagcaccga	
doeA-L arm-F	gagtcggtgctttttttgaacccggatatccagcgtacgaccg	
doeA-L arm-R	ctgtctaaacaatacaagggacatac	
doeA-R arm-F	tatgtcccttgtattgtttagacagcgggcaactcgccatca	
doeA-R arm-R	cagggttttcccagtcacgacggctcgcccatgccagcatc	
gRNA-doeA-F	tagtgcgaaatggcgtatcgtccc	
gRNA-doeA-R	aaacgggacgatacgccatttcgc	
gRNA-F	ctagggtctcaactagtattatacctaggactgagctagctgt	

gRNA-R	ctagggtctcagttttagagctagaaatagcaagttaaaataaggctagt	
ectD-L arm-F	gagtcggtgcttttttgaacccgggcttttgctatttcccaaggatg	Construction of pQ133-D
ectD-L arm-R	tttaatcatacaaggagtggattagtctaacgtatcactagtggtatgtatgaaa	
ectD-R arm-F	ctaatccactccttgtatgattaaatttcagatcaata	
ectD-R arm-R	cagggttttcccagtcacgacggctgagcgtctcagaagctgtac	
gRNA-ectD-F	tagtttaaccaccgcatcacgacg	
gRNA-ectD-R	aaaccgtcgtgatgcggtggttaa	
G4-backbone-F	cttgccatctgttttcttgcaagatctcaccttcatttccttatgctgaacacg	Construction of pQ41-ABC
G4-backbone-R	atcctgggacgatacgccatttcgctgatcgatgtgctcggaaggtg	
G4-ectABC-F	gcgaaatggcgtatcgtcccaggatgagtacgccaataacaccttttac	
G4-ectABC-R	ttcagcataaggaaatgaaggtgagatcttgcaagaaaacagatggcaag	
Porin-ectABC-L arm-F	gagtcggtgcttttttgaacccggccataaaccgtggtgacca	
Porin-ectABC-L arm-R	tgatcgatgtgctcggaag	
Porin140-F	atccaccttccgagcacatcgatcaatgcctccacaccgct	
Porin140-R	gcgtactcatctagtatttctcctctttctctagtaactcttaaacaaaattatttgtagag	
Porin-ectABC-R arm-F	atgagtacgccaataacacctttta	
Porin-ectABC-R arm-R	cagggttttcccagtcacgacggctgaaatattcacggctgtttagaggg	
G7-backbone-F	ggtgatatagagtgtatcgcgcaaagtt	Construction of pQ44-LysC
G7-backbone-R	ccttgatagtctcgaatcttccgacc	
Porin226-F	gtcggaagattcgagactatcaaggatgcctccacaccgctc	
Porin226-R	ctagtatttctcctctttctctagtaactcttaaacaaaattat	
Porin-lysC-F	tactagagaaagaggagaaatactagatggcc	
Porin-lysC-R	tttgcgcgatacactctatatcacctataaacgcagaaaggcccacc	
Porin58-F	aggttatctcgctgcccgacacttaatgcctccacaccgctc	Construction of pQ133-Asd-58/183
Porin58-R	catctagtatttctcctctttctctagtaactc	
Porin-asd-F	tactagagaaagaggagaaatactagatgttgaaag	
Porin-asd-R	caatagttatactgtataagataattataaacgcagaaaggcccacc	
G4-WT-F	tagctcagagagatcagcccatattacg	Colony PCR for both gene insertion and deletion
G4-WT-R	gctcaatacacggatcttatcggg	
Insertion-ectABC-R	tcgccttgcacggtttcaacaa	

G7-WT-F	tgggcacggctaaataccg
G7-WT-R	agcagcgcccaggtgaaa
Insertion-lysC-R [c]	atttgtagaggctgtttcgtcctcacg
G43-WT-F	ttcagcgacgcgcgcaatat
G43-WT-R	accactaaggctgcagcgc
Insertion-LuxR-R	gactctcttccgggcgctatcat
doeA-KO-F [d]	tgctgttgagtcttgtcgtctaaaacgt
doeA-KO-R [d]	aggtcgatcctgtggccca
doeA-WT-F [e]	cgcggaaaattggccagggtt
doeA-WT-R [e]	tatgccagaccgcggctgg
ectD-KO-F [d]	atcatatcggcaccttccc
ectD-KO-R [d]	gtgagcttataaaggtttctggtg
ectD-WT-F [e]	agctaggacgctgacttggag
ectD-WT-R [e]	atgacagtcagcaacaaccctctta

[a] F: forward design of primers (top strand); R: reverse design of primers (bottom strand);

[b] The names of plasmids can be found in 'plasmids' section of Supplementary Table 1;

[c] Primer sequences are designed in the *porin* promoter region used for PCR test of *asd* and *lysC* genes;

[d] Primer design of F_1/R_1 for *doeA* and *ectD* gene knockout refered to Fig. 4a;

[e] Primer design of F_2/R_2 for *doeA* and *ectD* gene knockout refered to Fig. 4a.

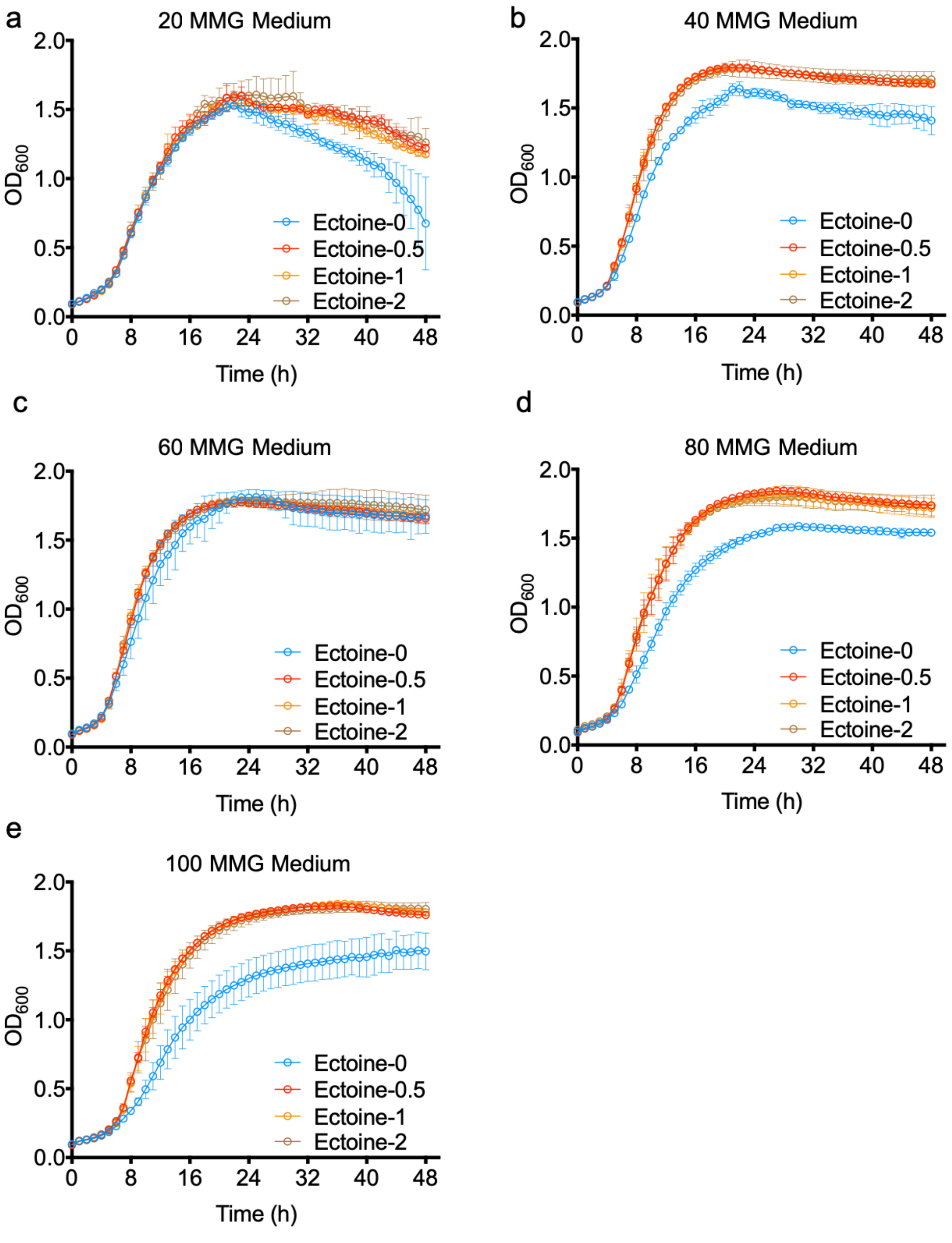

Supplementary Figure 1. Cell growth in a MM medium in the presecnce of different NaCl and ectoine concentrations, respectively.

Cell growth of wild type *H. bluephagenesis* TD1.0 cultured in 96-well plates in the MMG medium supplemented with 20 (**a**), 40 (**b**), 60 (**c**), 80 (**d**) and 100 (**e**) g L^{-1} NaCl, respectively, in the presence of various ectoine concentrations (0, 0.5, 1 and 2 g L^{-1}), respectively. OD_{600} was online monitored by a plate reader. Data are presented as mean values, error bars represent standard deviations (SD); n=3 biologically independent samples. Source data are provided as a Source Data file.

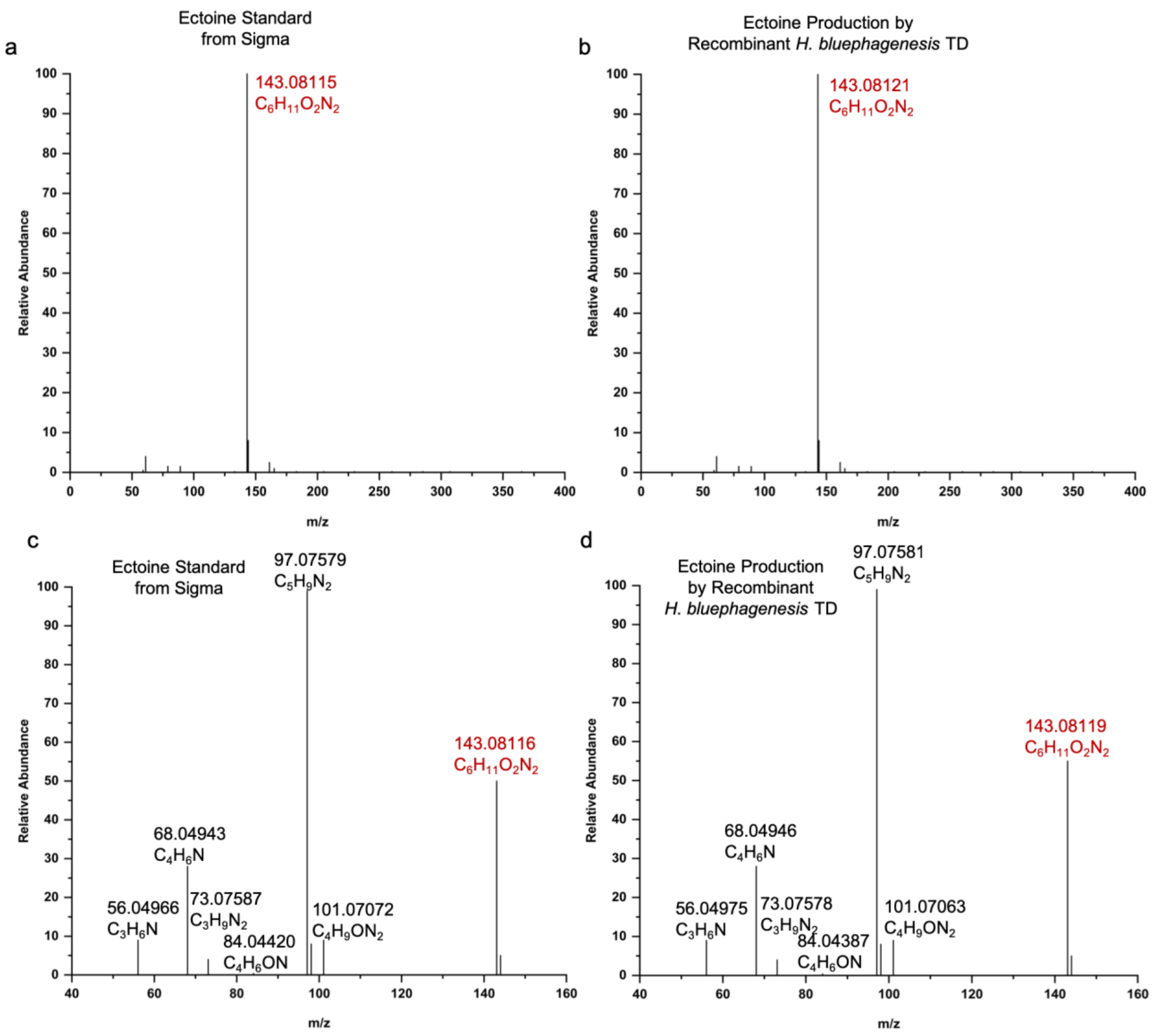

Supplementary Figure 2. LC-MS/MS characterization of ectoine produced by *H. bluephagenesis* TD1.0.

(**a**) and (**c**) represent LC-MS analytical results of an ectoine standard, (**b**) and (**d**) are ectoine produced by *H. bulephagenesis* TD1.0 grown in shake flask cultures, respectively. The molecular ion peak of ectoine shown in (**a**) and (**b**) is 143.08 (highlighted in red). (**c**) and (**d**) indicate the LC-MS/MS analytical results of the most abundant molecular ions from MS, the mass-to-charge ratio (m/z) at 143.08 represents the characteristic peak of ectoine (highlighted in red). Source data are provided in the Source Data file.

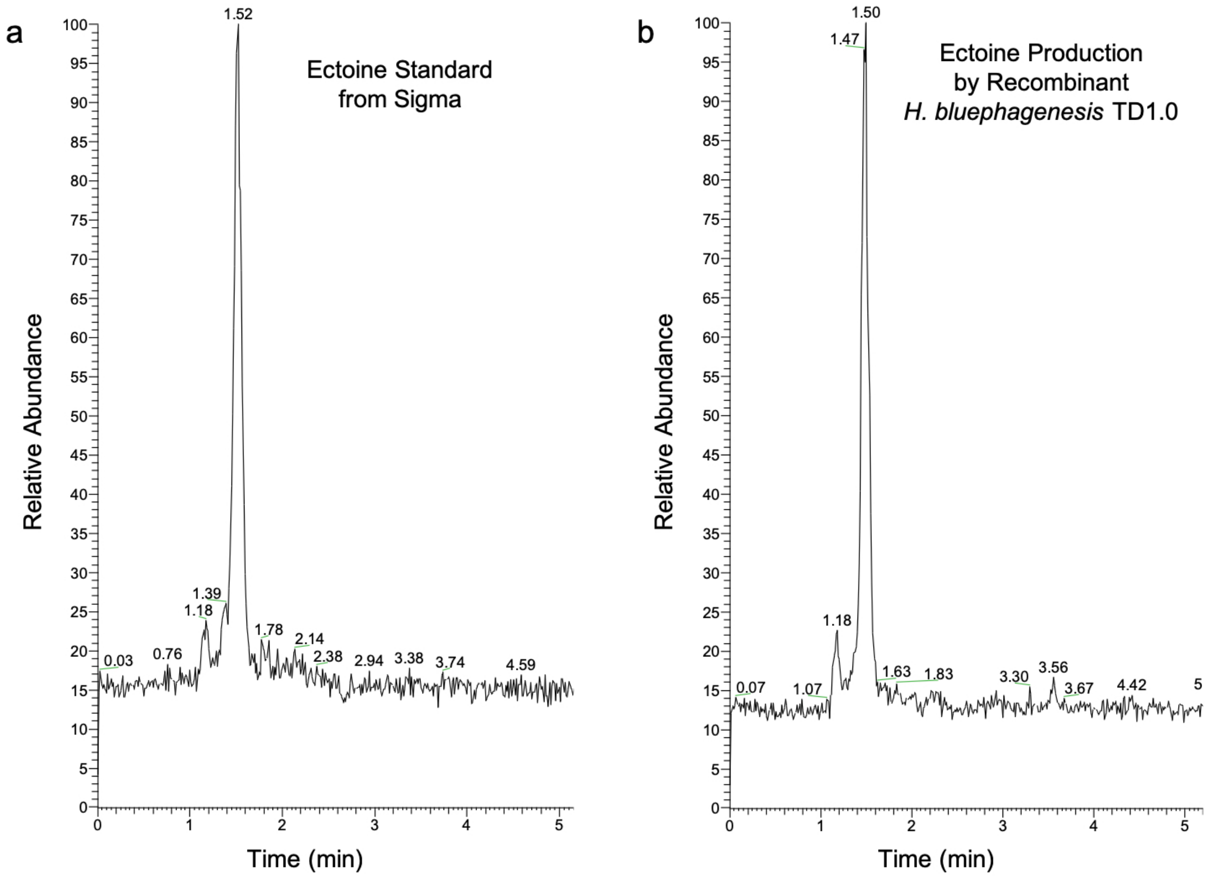

Supplementary Figure 3. HPLC analysis of ectoine produced by *H. bluephagenesis* TD1.0. (**a**) HPLC spectrum of ectoine standard, and (**b**) HPLC spectrum of ectoine produced by *H. bluephagenesis* TD1.0 in the supernatant of cultural broth after cell lysis. The retention time of target compound is approximately 1.5 min. Source data are provided as a Source Data file.

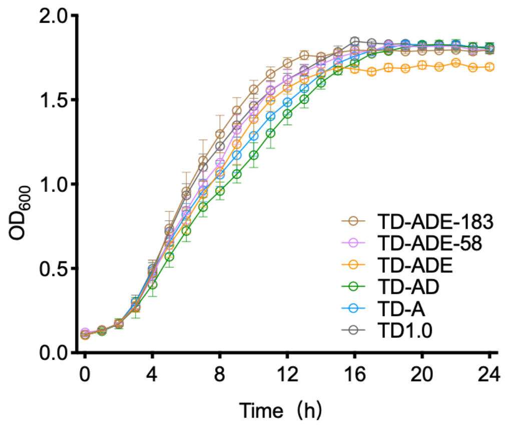

Supplementary Figure 4. Growth of chromosomally engineered *H. bluephagenesis* TD1.0 in a 60LB medium.

Online monitoring the growth (OD_{600}) of the chromosomally engineered strains, including *H. bluephagenesis* TD1.0 as the control, and its derivates, TD-A, TD-AD, TD-ADE, TD-ADEL-58 and TD-ADEL-183 cultured in the 60LB medium in a 96-well plate for 24 h. Data are presented as mean values, error bars represent standard deviations (SD); n=5 biologically independent samples. Source data are provided as a Source Data file.

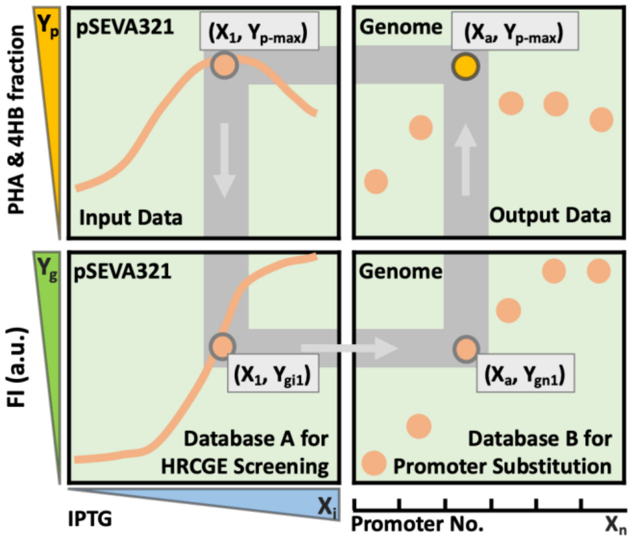

Supplementary Figure 5. Transcriptional tuning on chromosome of *H. bluephagenesis* TD01.

Transcriptional tuning design of GFP-mediated transcriptional mapping. Input data: represented the yield of target product (Y_p) with the same IPTG induction range under the HRCGE plasmid based system in 500-mL shake flasks. Database composed of the characterization information of induced systems (database A) and promoter mutants (database B). The reporter sGFP characterized with fluorescence intensity (Y_g) was used as global variables for transcriptional levels of various IPTG induction concentrations (X_i) or promoter mutants (X_n) with a wide range of promoter activity in plasmid- or chromosome-based systems. Output data: the maximum yield of the target product on chromosome-based system generated by the guidance of IPTG concentrations (X_1) leading to the maximum target production yield ($Y_{p\text{-}max}$). The promoter mutant screening for genomic integration was carried out subsequently followed by the FI value (Y_{gi1}) at IPTG concentration X_1. The promoter mutant X_a from the promoter mutants that performed the most similar FI value with Y_{gi1} (Y_{gn1}) having an equipotential transcriptional level compared with the IPTG induction concentration X_1 in induced systems, was recognized to have the best performance on target production yield ($Y_{p\text{-}max}$) in chromosome-based system. Figure and caption here are from Ye *et al.* for clearer illustration of GFP-mediated transcription mapping approach [7]. Reprinted from *Metabolic Engineering*, 57, Jianwen Ye, Dingkai Hu, Jin Yin, Wuzhe Huang, Ruijuan Xiang, Lizhan Zhang, Xuan Wang, Jianing Han, Guo-Qiang Chen, Stimulus response-based fine-tuning of polyhydroxyalkanoate pathway in *Halomonas*, 85-95, Copyright (2020), with permission from Elsevier.

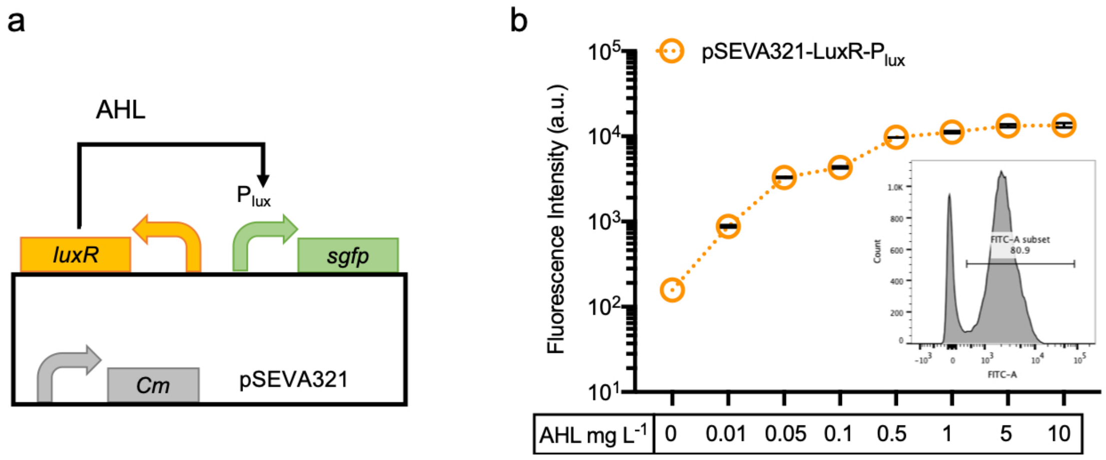

Supplementary Figure 6. Coarse characterization of LuxR-AHL inducible system on plasmid-based expression system in *H. bluephagenesis* TD1.0.

(**a**) Construction of luxR-AHL inducible system, the reporter gene (*sgfp*) controlled by promoter P_{lux} was used to characterize the transcriptional strength in the presence of various AHL concentrations, respectively. (**b**) Coarse characterization of the LuxR-AHL inducible system on plasmid-based expression system in *H. bluephagenesis* TD1.0 by recording the positive fluorescent cells (FITC-A subset) via flow cytometer analysis. A gating strategy was used to generate 'FITC-A subset' by substracting the negative cell counts (*y*-axis: cell count; *x*-axis: fluorescence intensity of FITC). Data are presented as mean values, error bars represent standard deviations (SD); n=3 biologically independent samples. Source data underlying Supplementary Fig. 6b are provided as a Source Data file.

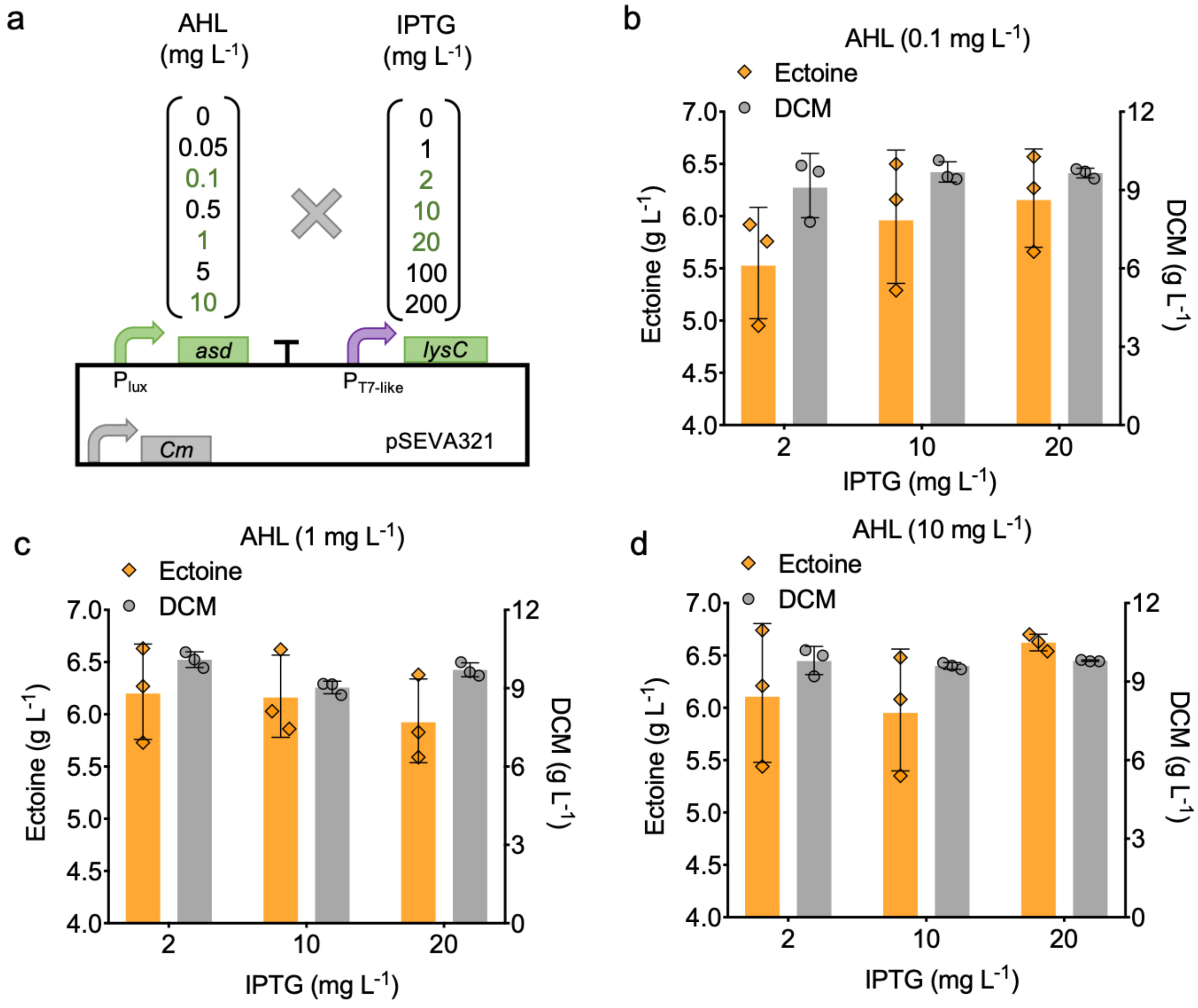

Supplementary Figure 7. Fine-tuning of *asd* and *lysC* genes based on two inducible systems in TD-ADE simultaneously.

(**a**) Transcriptional tuning design of *asd* and *lysC* gene, controlled by luxR-AHL and T7-like induced systems, respectively, for enhanced production of ectoine in the *ectABC* fine-tuned strain, TD-ADE, derived from *H. bluephagenesis* TD1.0. (**b**), (**c**) and (**d**) are the fermentation results of ectoine titers and DCM in a 60MMU5 medium in the presence of different combinatorary dosage of AHL and IPTG. 60MMU5 medium was derived from 60MMG medium supplemented with 5 g L^{-1} urea (see methods). Data are presented as mean values, error bars represent standard deviations (SD); n=3 biologically independent samples. Source data underlying Supplementary Fig. 7b-d are provided as a Source Data file.

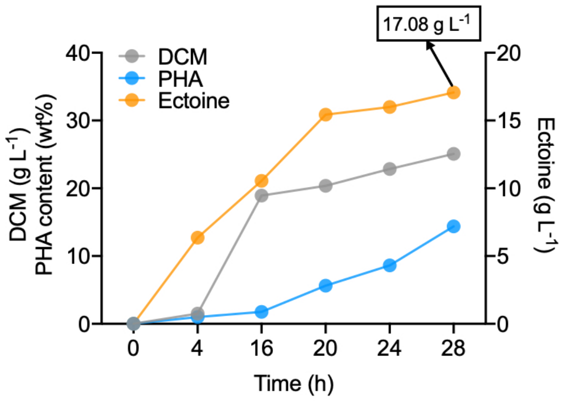

Supplementary Figure 8. Necessity analysis of gene *asd* and *lysC* tuning for ectoine production in a 7 L lab-scale fermentor.

Evaluation of growth, PHB and ectoine accumulation by recombinant strain, TD-ADE, derived from *H. bluephagenesis* TD1.0, harboring the fine-tuned expression of *ectABC* cluster on chromosome. Data were generated from one fed-batch fermentation under open unsterile condition without means and error bars. Source data are provided as a Source Data file.

Supplementary References

1. Simon R, Priefer U & Pühler A. A broad host range mobilization system for *in vivo* genetic engineering: transposon mutagenesis in gram negative bacteria. *Nat. Biotechnol.* **1**, 784-791 (1983).

2. Tan D, Xue YS, Aibaidula G & Chen GQ. Unsterile and continuous production of polyhydroxybutyrate by *Halomonas* TD01. *Bioresour. Technol.* **102**, 8130-8136 (2011).

3. Zhao H, *et al.* Novel T7-like expression systems used for *Halomonas*. *Metab. Eng.* **39**, 128-140 (2017).

4. Silva-Rocha R, *et al.* The standard european vector architecture (SEVA): a coherent platform for the analysis and deployment of complex prokaryotic phenotypes. *Nucleic Acids Res.* **41**, D666-675 (2013).

5. Qin Q, *et al.* CRISPR/Cas9 editing genome of extremophile *Halomonas spp*. *Metab. Eng.* **47**, 219-229 (2018).

6. Zeng W, *et al.* Rational design of an ultrasensitive quorum-sensing switch. *ACS Synth. Biol.* **6**, 1445-1452 (2017).

7. Ye J, *et al.* Stimulus response-based fine-tuning of polyhydroxyalkanoate pathway in *Halomonas*. *Metab. Eng.* **57**, 85-95 (2020).

8. Shen R, *et al.* Promoter engineering for enhanced P(3HB-*co*-4HB) production by *Halomonas bluephagenesis*. *ACS Synth. Biol.* **7**, 1897-1906 (2018).
