# Supplementary material for: Rational flux-tuning of Halomonas bluephagenesis for co-production of bioplastic PHB and ectoine
Source: Nat Commun. 2020 Jul 3;11:3313. doi: 10.1038/s41467-020-17223-3 (PMC7334215; doi:10.1038/s41467-020-17223-3)
Supplement: Supplementary file 3 — Reporting Summary [file 41467_2020_17223_MOESM3_ESM.pdf]

nature research

Corresponding author(s): Guo-qiang Chen
Jianwen Ye

Last updated by author(s): Jun 2, 2020

Reporting Summary

Nature Research wishes to improve the reproducibility of the work that we publish. This form provides structure for consistency and transparency in reporting. For further information on Nature Research policies, see Authors & Referees and the Editorial Policy Checklist .

Statistics

For all statistical analyses, confirm that the following items are present in the figure legend, table legend, main text, or Methods section.

n/a	Confirmed	
☐	☒	The exact sample size (*n*) for each experimental group/condition, given as a discrete number and unit of measurement
☐	☒	A statement on whether measurements were taken from distinct samples or whether the same sample was measured repeatedly
☒	☐	The statistical test(s) used AND whether they are one- or two-sided<br>*Only common tests should be described solely by name; describe more complex techniques in the Methods section.*
☒	☐	A description of all covariates tested
☒	☐	A description of any assumptions or corrections, such as tests of normality and adjustment for multiple comparisons
☐	☒	A full description of the statistical parameters including central tendency (e.g. means) or other basic estimates (e.g. regression coefficient) AND variation (e.g. standard deviation) or associated estimates of uncertainty (e.g. confidence intervals)
☒	☐	For null hypothesis testing, the test statistic (e.g. *F*, *t*, *r*) with confidence intervals, effect sizes, degrees of freedom and *P* value noted<br>*Give P values as exact values whenever suitable.*
☒	☐	For Bayesian analysis, information on the choice of priors and Markov chain Monte Carlo settings
☒	☐	For hierarchical and complex designs, identification of the appropriate level for tests and full reporting of outcomes
☒	☐	Estimates of effect sizes (e.g. Cohen's *d*, Pearson's *r*), indicating how they were calculated

Our web collection on statistics for biologists contains articles on many of the points above.

Software and code

Policy information about availability of computer code

Data collection	The cell growth data were collected using a micro-plate reader (Varioskan Flash, Thermo Scientific), and the other data were collected by commercial equipments and softwares shown in Methods and Materials section.
Data analysis	Genetic design and sequence reading were performed by Snap Gene (v2.3.2). Flow cytometer data were analyzed by FlowJo v7.6 software. All data were analyzed and graphed by GraphPad Prism8 and/or Microsoft 2016 (Excel, Word, PowerPoint).

For manuscripts utilizing custom algorithms or software that are central to the research but not yet described in published literature, software must be made available to editors/reviewers. We strongly encourage code deposition in a community repository (e.g. GitHub). See the Nature Research guidelines for submitting code & software for further information.

Data

Policy information about availability of data

All manuscripts must include a data availability statement. This statement should provide the following information, where applicable:

- Accession codes, unique identifiers, or web links for publicly available datasets
- A list of figures that have associated raw data
- A description of any restrictions on data availability

The authors declare that source data processed for figure generation in this study are available within the paper and its Supplementary Information files. The source data underlying Figs. 2a-b, 2d, 3b-d, 4a-c, 5b-c, 6b-d, 6f-g, 7a-b, as well as Supplementary Figs. 1a-e, 2a-d, 3a-b, 4, 6b, 7b-d and 8 are provided as a Source Data file. Plasmids used in this study are deposited in Source Data file. The datasets generated and analyzed during the current study are available from the corresponding authors upon request. A reporting summary for this Article is available as a Supplementary Information file.

Field-specific reporting

Please select the one below that is the best fit for your research. If you are not sure, read the appropriate sections before making your selection.

☒ Life sciences ☐ Behavioural & social sciences ☐ Ecological, evolutionary & environmental sciences

For a reference copy of the document with all sections, see nature.com/documents/nr-reporting-summary-flat.pdf

Life sciences study design

All studies must disclose on these points even when the disclosure is negative.

Sample size
No sample size calculation and statistical test was performed in this study. The sample size of experiments is determined at 3 (biological independent replicate) except the cell growth study (5 biological independent replicate) in a 96-well plate and fad-batch studies in a 7-L fermenter (one batch) to assess the comprehensive performance of target strains presented as Mean value with standard deviation. Each sample was measured once.

Data exclusions
No data exclusions.

Replication
Each sample included 3 biological replicates (5 replicates in Supplementary Figure 4 for cell growth study of recombinant strains) except the fad-batch studies in a 7-L fermenter (one batch). Experimental data were presented as Mean ± S.D. value (clear distribution of data points are shown in histogram plots).

Randomization
No randomization was performed in the study, since all replicates are processed to be test or measured in this study.

Blinding
Blinding was not performed in the study. Because the experiments did not involve any animals or human participants. For cell culturing, seed cells are cultured from single colonies and then inoculated into different 500-mL conical flasks or 7-L bioreactor described in the manuscript. And the results are objective.

Reporting for specific materials, systems and methods

We require information from authors about some types of materials, experimental systems and methods used in many studies. Here, indicate whether each material, system or method listed is relevant to your study. If you are not sure if a list item applies to your research, read the appropriate section before selecting a response.

Materials & experimental systems

n/a	Involved in the study
☒	☐ Antibodies
☒	☐ Eukaryotic cell lines
☒	☐ Palaeontology
☒	☐ Animals and other organisms
☒	☐ Human research participants
☒	☐ Clinical data

Methods

n/a	Involved in the study
☒	☐ ChIP-seq
☐	☒ Flow cytometry
☒	☐ MRI-based neuroimaging

Flow Cytometry

Plots

Confirm that:

☒ The axis labels state the marker and fluorochrome used (e.g. CD4-FITC).

☒ The axis scales are clearly visible. Include numbers along axes only for bottom left plot of group (a 'group' is an analysis of identical markers).

☒ All plots are contour plots with outliers or pseudocolor plots.

☒ A numerical value for number of cells or percentage (with statistics) is provided.

Methodology

Sample preparation
Cells carrying target expression vessels were plated on 60 LB agar plate added with relevant antibiotics for 16-20 h of incubation at 37°C. Subsequently, single colony was inoculated into 1 mL 60LB medium for 12 h cultivation in 96-deep-well plate covered with sealing films (BF-400-S; Thermo-Shaker, Aosheng, 37°C, 1000 rpm). Then, 5 μL cell culture was inoculated into a new plate supplemented with appropriate antibiotics in presence of a spectrum of inducer. After 12 h incubation, cell cultures were diluted 250-folds using phosphate-buffered saline solution (PBS) for flow cytometry analysis.

Instrument	Flow cytometer (LSRFortessa4, BD bioscience, USA)
Software	FlowJo (v7.6) software was used to process the raw data for obtaining mean value of fluorescence intensity and percentage of fluorescent positive cells.
Cell population abundance	Cell cultures after 12 h cultivation were diluted 250-folds using phosphate-buffered saline solution (PBS) and recorded by flow cytometer (LSRFortessa4, BD bioscience, USA) at the rate of 0.5 µL s^-1 for 20 seconds with at least 30,000 cell counts were captured.
Gating strategy	Intracellular sfGFP protein was excited under 488 nm, and cells were captured on the signal channels of FITC(voltage 440 V), FSC (forward scatter, voltage 440 V) and SSC (side scatter, voltage 260 V). All captured events were used for fluorescence analysis.

☒ Tick this box to confirm that a figure exemplifying the gating strategy is provided in the Supplementary Information.
