# Supplementary material for: Rational flux-tuning of Halomonas bluephagenesis for co-production of bioplastic PHB and ectoine
Source: Nat Commun. 2020 Jul 3;11:3313. doi: 10.1038/s41467-020-17223-3 (PMC7334215; doi:10.1038/s41467-020-17223-3)
Supplement: Supplementary file 4 — Description of Additional Supplementary Files [file 41467_2020_17223_MOESM4_ESM.pdf]

Description of Additional Supplementary Files

File name: Supplementary Data 1

Description: Sequence of Plasmid No. 01 p321-Ptac-ectABC

File name: Supplementary Data 2

Description: Sequence of Plasmid No. 02 p321-PJ23110-luxR-Plux-sfgfp

File name: Supplementary Data 3

Description: Sequence of Plasmid No. 03 p321-Plux-sfgfp

File name: Supplementary Data 4

Description: Sequence of Plasmid No. 04 p321-PJ23110-lacI-PT7-like-sfgfp

File name: Supplementary Data 5

Description: Sequence of Plasmid No. 05 p321-PJ23110-lacI-PT7-like-ectABC

File name: Supplementary Data 6

Description: Sequence of Plasmid No. 06 p321-PT7-like-lysC-Plux-asd

File name: Supplementary Data 7

Description: Sequence of Plasmid No. 07 pQ133-LuxR

File name: Supplementary Data 8

Description: Sequence of Plasmid No. 08 pQ133-A

File name: Supplementary Data 9

Description: Sequence of Plasmid No. 09 pQ133-D

File name: Supplementary Data 10

Description: Sequence of Plasmid No. 10 pQ41-ABC

File name: Supplementary Data 11

Description: Sequence of Plasmid No. 11 pQ44-LysC

File name: Supplementary Data 12

Description: Sequence of Plasmid No. 12 pQ133-Asd-58

File name: Supplementary Data 13

Description: Sequence of Plasmid No. 13 pQ133-Asd-183
