# Supplementary material for: Rational flux-tuning of Halomonas bluephagenesis for co-production of bioplastic PHB and ectoine
Source: Nat Commun. 2020 Jul 3;11:3313. doi: 10.1038/s41467-020-17223-3 (PMC7334215; doi:10.1038/s41467-020-17223-3)
Supplement: Supplementary file 2 — Peer Review File [file 41467_2020_17223_MOESM2_ESM.pdf]

Reviewer #1 (Remarks to the Author):

Ma and colleagues engineered ectoine overproduction in Halomonas bluephagenesis with/without polyhydroxyalkanoate overproduction. Halomonas synthesizes ectoine upon hyperosmotic stress. Overexpression of the endogenous ectABC operon allowed for ectoine overproduction, deletion of ectoine converting genes (doeA, ectD) and overexpression of precursor genes improved production. Open bioreactors were used to produce 28 g/L ectoine. As expected, nitrogen limitation interfered with ctoine production which was reduced to 8 g/L since carbon was lost for polyhydroxybutyrate production.
Major criticism:
Conceptionally, it is not clear why the author stress coproduction? Is ectoine to be used in bioplastics? What would be an advantage of ectoine + PHB coproduction as compared to individual production of ectoine and PHB at much higher levels and mixing separately produced PHB and ectoine?
Bioreactor data are from a preliminary strain (TD1.0 according to the caption). Authors improved this strain (s. strain list TD-ADE-58). Why have they not compare TD1.0 with TD-ADE-58? These data should be shown.
Authors may delete genes for PHB biosynthesis to boost ectoine production.
To deduce the function of the conserved ectABC encoded proteins, authors may construct and analyse deletion mutants.

Authors shall calculate ectoine yields on glucose and volumetric productivities.

All figure legends: were replicate cultivations analysed or sample quantification was done in replicates: explain here
Figure 7 legend should contain information how many biological replicates were analysed and explain meaning of error bars. If no replicates were performed indicate this fact.

Suppl table:
List of genes: give gene IDs from Genbank instead of a ref. or "this study"

Reviewer #2 (Remarks to the Author):

In the manuscript, the authors build on their previous work with a halophilic bacterium, engineering it to produce high concentrations of ectoine, a solute produced by this class of organisms. The authors verify that their chosen organism, Halomonas bluephagenesis, contains the ectoine biosynthetic pathway, and perform a number of genetic manipulations along with medium formulation to achieve a maximum titer of 28 g/L in an "open" fermentation. Lastly, they demonstration the co-production of ectoine with the biopolymer PHB, albeit at lower titers. The manuscript is relatively straightforward, though some parts can be a bit confusing. The titers achieved are impressive, although the methods employed have all been previously reported. The following specific comments are intended to help strengthen the manuscript:

1. Line 54 – I do not believe it is fair to call a sterile bioprocess "complicated" as it implies that this is not the current standard in the biotech industry. Non-sterile processes can also be "complicated."

2. Lines 154-156 – The medium named "60MME" has 60 g/L NaCl by definition. Therefore, why was there a "factorial analysis" to find the optimized concentration?

3. Line 157, Figure 3c – 60 g/L was noted to give the "highest" concentration at 2.82 g/L. Is this really significantly different from what was achieved with 80 g/L?

4. Lines 165-167 – It should be noted that the value of 2.83 g/L achieved with urea matches but

doesn’t exceed that obtained with aspartate supplementation. As written, it is easy for the reader to mistake this as a significant improvement.

5. Lines 212-214 – What was the “stronger promoter” that could not be constructed?

6. Line 221 – It is not “remarkable” that the negative control strain is so low – that’s why it is the negative control!

7. Line 232 – I don’t agree with the use of the word “biosensor” here. Biosensors respond to specific metabolites or ligands and regulate gene expression in response. These are simply chemically-inducible promoters, which are both very useful and well-established.

8. Line 253 – Most “induction combinatory groups had enhanced production” over what? Only the induced results are shown in Figure 6f, so it’s not clear what the reference point should be.

9. Line 259-262 – The first two sentences are a bit confusing. I think it would help to include the promoter that *did* work for lysC. As written, the reader could be left thinking that lysC was not overexpressed at all, only asd.

10. Line 262 – I think “6h” should be “6g” since there is no panel “6h”.

11. Line 328 – Please use “quorum-sensing-based” rather than “sensor-based” since this is more descriptive.

12. Line 342 – The authors are free to ignore this suggestion, but I would note that PHB is derived from acetyl-CoA rather than acetoacetyl-CoA. It is one step further back, but is a much more well-known metabolic intermediate.

13. Methods – In the initial description of the medium, yeast extract is listed as an ingredient. In many places that follow, this becomes just “yeast” which I assume is a mistake.

14. Figure 2a – The heat map with black is very hard to read. Since the rest of the figure is in color, I strongly suggest using a color scale to represent the data.

15. Figure 3a – A very short description of the “T7-like” system, even just specifying what MmP1 is, would be helpful.

16. Figure 4, lines 57-59 – The description of the strain names makes it seem like TD1.0 does NOT contain the ectoine synthesis plasmid. Is this correct?

17. Figure 6, panel g legend – The order of 60MMU and 60MMU5 in the legend should match the order the data are presented (i.e., 5 g/L of urea is first in the figure).

18. Figure S7 is not referenced anywhere in the main text.

Language errors (these are just a few – please proofread carefully):

19. Lines 76-79 – This sentence does not read quite right. Perhaps it should say “…were successfully used to direct metabolic fluxes…”?

20. Line 88 – “engineering” should be “engineer”

21. Line 98 – “depended” should be “dependent”

22. Line 120 – “heterogeneous” should be “heterologous” (and in other places, too)

23. Line 120 – ectoine is misspelled

24. Line 155 – “mediums” should be “media”

25. Line 179 – “double checked” should be “confirmed”

26. Line 199 – I don’t think “variable” is the right word – “reporter” is more standard.

27. Line 262 – Is “also” correct or should this be “almost”?

28. Lines 268-270 – If I am reading Fig S4 correctly, the 96-well plate cultures were done. That means “should be” should be “were”.

29. Line 397 – “Qiagen” is misspelled

30. Line 456 – “whatever” should be “whenever”

31. Figure S4, line 77 – remove “respectively”

32. Figure S5, line 90 – “guildance” should be “guidance”

Reviewer #3 (Remarks to the Author):

Dear Authors,

I reviewed your highly intriguing manuscript, which constitutes the next elaboration on Halomonas bluephagenesis, the prototype PHA production strain used for PHA production in the context of “Next Generation Industrial Biotechnology” (NGIB), which comprises the cultivation of engineered robust, powerful production strains under minor sterility precautions in a cost-efficient manner.
The manuscript at hand deals with the co-production of PHA (homopolyester PHB) and the osmo-solute ectoine; while PHB accrues as intracellular product, ectoine can be harvested as extracellular product via “bacterial milking” in hyposaline medium.
A new ectoine biosynthesis pathway was constructed in H. bluephagenesis by fine-tuning the transcription levels of involved genes using two biosensor responsive systems. In fed-batch cultivation, the genetically engineered bacterial construct was able to produce a total of 8 g/L and 32 g/L cell dry mass harboring 75 wt.-% PHB within 28h of cultivation.
Indeed, the work contributes to the development of versatile microbial “working horses”, which are capable of co-producing different marketable bioproducts under inexpensive cultivation conditions, which is the pre-condition for industrial-scale feasibility.
The work is scientifically sound, the experiments are well designed and reproducible for peers, and the conclusions are well supported by the elaborated data. For sure, the work is of interest for the scientific community active in this field.
Only some minors should be addressed in order to further improve the submission:
A) Abstract: no need to abbreviate the species name in brackets at first occurrence (line 30-31)
B) Title: “Rational Flux-Tuning Halomonas bluephagenesis for…” should rather be “Rational Flux-Tuning of Halomonas bluephagenesis for…”
C) Polymer name: “polyhydroxybutyrate” should be “poly(3-hydroxybutyrate)” (think about poly(4-hydroxybutyrate)”. In abstract and text.
D) Some English editing will welcome, e.g., line 46: “the most well-known”: better: “the best known”; line 51: “…, organ…”: “…, and organ…”; line 69: “saltless”: better: desalted”; line 88: “to engineering

H. bluephagenesis": "to engineer H. bluephagenesis"; line 102: "economy improvement": "economic improvement"; line 116-117: "..blast results…shows…": "..blast results…show…"; line 274: "Ectoine": "ectoine"; line 328: "It has to be note": "It has to be noted". Line 495: "Follwed by…..quantification": please, rephrase this sentence; it is not clear.

E) A minor: "L-aspartate" etc.: "L" should not be in capital letter, but, scientifically correct, in small caps.

F) Check style of references in text: e.g., line 50: "2, 3, 4, 5, 6, 7": I suppose, it should be "2-7" (also elsewhere in text)

G) Line 85: "by man bacteria": add "and archaea"; not only bacteria accumulate PHA!

H) Figure legend 1 (line 10) and elsewhere: "β-ketothiolase" today is "3-ketothiolase"; Figure 1: Check abbreviation: "P3HB" is "PHB" in the main text and legend! Line 9 in legend: "and" should not be in italics

Listed Responses to Reviewers of Ref: **NCOMMS-20-06233**

Title: **Rational Flux-Tuning of *Halomonas bluephagenesis* for Co-production of Bioplastic PHB and Ectoine**

We are grateful to the **three reviewers** for their efforts to handle and review our paper. After carefully studying the comments and suggestions, and following responses have been generated (highlighted in red color):

Comments from reviewers:

Reviewer #1 (Remarks to the Author):

Ma and colleagues engineered ectoine overproduction in *Halomonas bluephagenesis* with/without polyhydroxyalkanoate overproduction. *Halomonas* synthesizes ectoine upon hyperosmotic stress. Overexpression of the endogenous *ectABC* operon allowed for ectoine overproduction, deletion of ectoine converting genes (doeA, ectD) and overexpression of precursor genes improved production. Open bioreactors were used to produce 28 g/L ectoine. As expected, nitrogen limitation interfered with ctoine production which was reduced to 8 g/L since carbon was lost for polyhydroxybutyrate production.

Response: Thank you for your precise summary of our study.

Major criticism:

1. Conceptionally, it is not clear why the author stress coproduction? Is ectoine to be used in bioplastics? What would be an advantage of ectoine + PHB coproduction as compared to individual production of ectoine and PHB at much higher levels and mixing separately produced PHB and ectoine?

Response: Thank you for your questions. Since ectoine is an extracellular product formed during the log phase in the presence of rich nitrogen source, while PHB an intracellular one produced after the log phase under nitrogen limitation, coproduction of ectoine and PHB thus allows recovery of two products from broth and biomass, the process economy can be significantly improved.

2. Bioreactor data are from a preliminary strain (TD1.0 according to the caption). Authors improved this strain (s. strain list TD-ADE-58). Why have they not compared TD1.0 with TD-ADE-58? These data should be shown.

Response: In this study, bioreactor data of strain TD-ADE, a control strain, are presented in supplementary Fig. S8 for comparation to the results of strain TD-ADE-58 under the same conditions. Since the start host TD1.0 was grown very poorly due to the rich nitrogen medium with cumulative ammonia at pH=8.5, namely, TD1.0 did not grown well under the above conditions. Thus, this batch study could not compare results of strain TD1.0 with TD-ADE-58.

3. Authors may delete genes for PHB biosynthesis to boost ectoine production.

Response: Thank you for your good suggestion. Since the strain has multiple copies of PHB synthesis related genes including two *phaC* (2 copies), three *phaB* (3 copies) and five *phaA* (5 copies). Unless all of the genes are deleted, it is hard to completely eliminated PHB synthesis. However, PHB biosynthesis can be weakened to close to 10% storage from more than 80% (referred to cell dry weight) by a nitrogen rich medium instead of deleting genes for PHB synthesis, yet not significant improvement was observed for ectoine production (Fig. 3d).

4. To deduce the function of the conserved ectABC encoded proteins, authors may construct and analyse deletion mutants.

Response: It is difficult to delete any gene of *ectABC* operon in *Halomonas bluephagenesis* TD1.0, as ectoine is an essential molecule for its survival in a high salt concentration. However, in order to verify the function of proteins encoded by *ectABC*, heterologous expression of *ectABC* was carried out in *E. coli* to produce 2-4 g/L ectoine in the presence of various IPTG concentrations, respectively (Fig. 2d). This is a commonly used approach to confirm the function of *ectABC* operon as *E. coli* is not a natural ectoine producer.

5. Authors shall calculate ectoine yields on glucose and volumetric productivities.

Response: Thank you for your suggestion. The yield of ectoine on glucose was calculated to be approximately 0.21 g/g. The volumetric productivity was found to be 1.0 g/L/h during the fed-batch cultivation of strain TD-ADE-58. These two data have been added in the revised paper.

6. All figure legends: were replicate cultivations analysed or sample quantification was done in replicates: explain here

Response: Yes, all data were generated from three replicate cultivations, “n =3” indicates triplicate cultivations.

7. Figure 7 legend should contain information how many biological replicates were analysed and explain meaning of error bars. If no replicates were performed indicate this fact.

Response: Thank you for your suggestion. There was one biological replicate shown in Figure 7 (from a batch fermentation). Fermentor studies are mostly not conducted in parallel studies. Brief description has been added in the legend as suggested.

8. Suppl table:
List of genes: give gene IDs from Genbank instead of a ref. or “this study”

Response: Thank you. The gene IDs from Genebank has been added in the supplementary Table.

We are grateful to Reviewer #1 for the constructive suggestions and careful reading of our paper.

Reviewer #2 (Remarks to the Author):

In the manuscript, the authors build on their previous work with a halophilic bacterium, engineering it to produce high concentrations of ectoine, a solute produced by this class of organisms. The authors verify that their chosen organism, *Halomonas bluephagenesis*, contains the ectoine biosynthetic pathway, and perform a number of genetic manipulations along with medium formulation to achieve a maximum titer of 28 g/L in an "open" fermentation. Lastly, they demonstration the co-production of ectoine with the biopolymer PHB, albeit at lower titers. The manuscript is relatively straightforward, though some parts can be a bit confusing. The titers achieved are impressive, although the methods employed have all been previously reported. The following specific comments are intended to help strengthen the manuscript:

Response: Thank you for your very good summary of our study.

1. Line 54 – I do not believe it is fair to call a sterile bioprocess "complicated" as it implies that this is not the current standard in the biotech industry. Non-sterile processes can also be "complicated."

Response: Thank you for your comments. The word "Complicated" has been removed.

2. Lines 154-156 – The medium named "60MME" has 60 g/L NaCl by definition. Therefore, why was there a "factorial analysis" to find the optimized concentration?

Response: The NaCl concentration in the medium named "60MME" or "60MMU" was obtained and optimized based on the factorial analysis of NaCl concentrations from 20 to 100 g/L (Fig. 3c). 60 g/LNaCl concentration regarded as optimal for the strain (Tan et al 2011) was thus used for conceptional proof of ectoine production by recombinant *H. bluephagenesis* TD1.0.

3. Line 157, Figure 3c – 60 g/L was noted to give the "highest" concentration at 2.82 g/L. Is this really significantly different from what was achieved with 80 g/L?

Response: The titer of ectoine obtained at 80 g/L NaCl was close to that generated under 60 g/L, under which ectoine reached 2.82 g/L. However, a lower NaCl concentration is better than a higher one, especially for the scale-up purpose when issues such as material cost and waste water treatment should be considered.

4. Lines 165-167 – It should be noted that the value of 2.83 g/L achieved with urea matches but doesn't exceed that obtained with aspartate supplementation. As written, it is easy for the reader to mistake this as a significant improvement.

Response: Thank you for your suggestion. We have added comment to clear in the meaning: "This resulted in a 2.83 g/L ectoine produced by shake flask growing recombinant *H. bluephagenesis* harboring *ectABC* expression module induced by 20 mg/L IPTG (Fig. 3d), this is **close to the ectoine amount generated from cultures in 60MME medium**."

5. Lines 212-214 – What was the “stronger promoter” that could not be constructed?

Response: The “stronger promoter” means that a promoter with a fluorescence intensity (FI) value stronger than 50,000 (the FI of P_{140} is about 50,900). This could not be constructed in our strains. Since it was reported that expression levels of target modules on a plasmid-based system can be 9-folds higher than that on a chromosome-based system in *H. bluephagenesis* (Ye et al., 2020). Thus, construction of such high expression vessels in donor strains (*E. coli* S17-1) is still challenging based on previous genome editing tools (Qin et al., 2018), because the requirement of a high expression level of integrated modules usually cannot be constructed in donor (*E. coli* S17-1) cells due to the overload of target fluxes. The revision has been made in the text.

6. Line 221 – It is not “remarkable” that the negative control strain is so low – that’s why it is the negative control!

Response: The “remarkable” has been removed from the text and sentence rephrased to “…yet **much higher than that of the control** *H. bluephagenesis* TD-AD strain…”.

7. Line 232 – I don’t agree with the use of the word “biosensor” here. Biosensors respond to specific metabolites or ligands and regulate gene expression in response. These are simply chemically-inducible promoters, which are both very useful and well-established.

Response: Thank you for your correction. The sentence has been revised to “Two inducible promoters…” in the text.

8. Line 253 – Most “induction combinatory groups had enhanced production” over what? Only the induced results are shown in Figure 6f, so it’s not clear what the reference point should be.

Response: The sentence was rephrased to “The induction concentrations of IPTG and AHL did have a significant influence on ectoine production.”.

9. Line 259-262 – The first two sentences are a bit confusing. I think it would help to include the promoter that *did* work for lysC. As written, the reader could be left thinking that lysC was not overexpressed at all, only asd.

Response: Thank you for your suggestion. To avoid the confusion, the name of the strain TD-ADE-58/183 was rewritten as **TD-ADEL-**58/183 to indicate that the promoter did work for *lysC* gene.

10. Line 262 – I think “6h” should be “6g” since there is no panel “6h”.

Response: Yes, it should be “6 g”. Revision done.

11. Line 328 – Please use “quorum-sensing-based” rather than “sensor-based” since this is more descriptive.

Response: Thank you. Done!

12. Line 342 – The authors are free to ignore this suggestion, but I would note that PHB is derived from acetyl-CoA rather than acetoacetyl-CoA. It is one step further back, but is a much more well-known metabolic intermediate.
Response: Thank you. Done!

13. Methods – In the initial description of the medium, yeast extract is listed as an ingredient. In many places that follow, this becomes just "yeast" which I assume is a mistake.

Response: Thank you. The mistakes have been corrected.

14. Figure 2a – The heat map with black is very hard to read. Since the rest of the figure is in color, I strongly suggest using a color scale to represent the data.

Response: Thank you for your suggestion. The heat map has been revised to become a color scale one.

15. Figure 3a – A very short description of the "T7-like" system, even just specifying what MmP1 is, would be helpful.

Response: Thank you. A brief introduction of "T7-like" system has been added in the legend: "A T7-like promoter ($P_{T7\text{-like}}$) activated by the MmP1 polymerase, which is controlled by P_{tac} promoter and induced by IPTG, namely, the strong T7-like inducible system, was employed to control the expression of *ectABC* operon.".

16. Figure 4, lines 57-59 – The description of the strain names makes it seem like TD1.0 does NOT contain the ectoine synthesis plasmid. Is this correct?

Response: No, all of the three strains contain the ectoine synthesis plasmid. To avoid confusion, additional description has been added in the legend.

17. Figure 6, panel g legend – The order of 60MMU and 60MMU5 in the legend should match the order the data are presented (i.e., 5 g/L of urea is first in the figure).

Response: Thank you for your suggestion. The order in the sentence has been revised to "… 60MMU5 and 60MMU…" to match the figure from left to right.

18. Figure S7 is not referenced anywhere in the main text.

Response: Fig. S7 has now been referenced together with **Figs. 6d and 6f**.

Language errors (these are just a few – please proofread carefully):

19. Lines 76-79 – This sentence does not read quite right. Perhaps it should say "…were successfully used to direct metabolic fluxes…"?

Response: Thank you. Revision has been done.

20. Line 88 – "engineering" should be "engineer"

Response: Thank you. Revision done.

21. Line 98 – "depended" should be "dependent"

Response: Thank you. Revision done.

22. Line 120 – "heterogeneous" should be "heterologous" (and in other places, too)

Response: Thank you. Revision done.

23. Line 120 – ectoine is misspelled

Response: Thank you. Revision done.

24. Line 155 – "mediums" should be "media"

Response: Thank you. Revision done.

25. Line 179 – "double checked" should be "confirmed"

Response: Thank you. Revision done.

26. Line 199 – I don't think "variable" is the right word – "reporter" is more standard.

Response: Thank you. Revision done.

27. Line 262 – Is "also" correct or should this be "almost"?

Response: Thank you. Revision done.

28. Lines 268-270 – If I am reading Fig S4 correctly, the 96-well plate cultures were done. That means "should be" should be "were".

Response: Thank you. Revision done.

29. Line 397 – "Qiagen" is misspelled

Response: Thank you. “Qiagen” should be “Tiangen”

30. Line 456 – “whatever” should be “whenever”

Response: Thank you. Revision done.

31. Figure S4, line 77 – remove “respectively”

Response: Thank you. The “respectively” removed.

32. Figure S5, line 90 – “guildance” should be “guidance”

Response: Thank you. Revision done.

We are grateful to Reviewer #2 for the constructive suggestions and careful reading of our paper.

Reviewer #3 (Remarks to the Author):

Dear Authors,

I reviewed your highly intriguing manuscript, which constitutes the next elaboration on *Halomonas bluephagenesis*, the prototype PHA production strain used for PHA production in the context of “Next Generation Industrial Biotechnology” (NGIB), which comprises the cultivation of engineered robust, powerful production strains under minor sterility precautions in a cost-efficient manner.
The manuscript at hand deals with the co-production of PHA (homopolyester PHB) and the osmo-solute ectoine; while PHB accrues as intracellular product, ectoine can be harvested as extracellular product via “bacterial milking” in hyposaline medium.
A new ectoine biosynthesis pathway was constructed in *H. bluephagenesis* by fine-tuning the transcription levels of involved genes using two biosensor responsive systems. In fed-batch cultivation, the genetically engineered bacterial construct was able to produce a total of 8 g/L and 32 g/L cell dry mass harboring 75 wt.-% PHB within 28h of cultivation.
Indeed, the work contributes to the development of versatile microbial “working horses”, which are capable of co-producing different marketable bioproducts under inexpensive cultivation conditions, which is the pre-condition for industrial-scale feasibility.
The work is scientifically sound, the experiments are well designed and reproducible for peers, and the conclusions are well supported by the elaborated data. For sure, the work is of interest for the scientific community active in this field.
Only some minors should be addressed in order to further improve the submission:

Response: Thank you very much indeed for your very nice comments to our study.

A) Abstract: no need to abbreviate the species name in brackets at first occurrence (line 30-31)

Response: Thank you for your comments. The abbreviation was removed, and revision done in the abstract.

B) Title: "Rational Flux-Tuning *Halomonas bluephagenesis* for…" should rather be "Rational Flux-Tuning of *Halomonas bluephagenesis* for…"

Response: Thank you. Revision done.

C) Polymer name: "polyhydroxybutyrate" should be "poly(3-hydroxybutyrate)" (think about poly(4-hydroxybutyrate)". In abstract and text.

Response: Thank you. Revision done all over the paper.

D) Some English editing will welcome, e.g., line 46: "the most well-known": better: "the best known"; line 51: "…, organ…": "…, and organ…"; line 69: "saltless": better: desalted"; line 88: "to engineering H. bluephagenesis": "to engineer H. bluephagenesis"; line 102: "economy improvement": "economic improvement"; line 116-117: "..blast results…shows…": "..blast results…show…"; line 274: "Ectoine": "ectoine"; line 328: "It has to be note": "It has to be noted". Line 495: "Follwed by…..quantification": please, rephrase this sentence; it is not clear.

Response: Thank you for your careful reading and patience to find errors. Revisions have been done in the text. And the sentence of "Followed by…quantification" has been rephrased to "Then, the supernatants were filtrated once by 0.22 μm filter membrane for ectoine quantification using high-performance liquid chromatography".

E) A minor: "L-aspartate" etc.: "L" should not be in capital letter, but, scientifically correct, in small caps.

Response: Thank you for your suggestion. "L-…" in capital has been revised to "L-…" in small caps.

F) Check style of references in text: e.g., line 50: "2, 3, 4, 5, 6, 7": I suppose, it should be "2-7" (also elsewhere in text)

Response: Thank you. Revisions done.

G) Line 85: "by man bacteria": add "and archaea"; not only bacteria accumulate PHA!

Response: Thank you for your suggestion. Revision done.

H) Figure legend 1 (line 10) and elsewhere: "β-ketothiolase" today is "3-ketothiolase"; Figure 1: Check abbreviation: "P3HB" is "PHB" in the main text and legend! Line 9 in legend: "and" should not be in italics.

Response: Thank you. Revisions done.
Once again, we thank three reviewers and the editor for their precious time and energy to help improve the quality of our paper.

REVIEWERS' COMMENTS:

Reviewer #1: unavailable.

Reviewer #2 (Remarks to the Author):

The authors have done a nice job responding to my (somewhat lengthy comments). I only have 2 points that I don't feel were adequately addressed but which can be quite easily:

1. Original comment, now Lines 153-155 – "The medium named "60MME" has 60 g/L NaCl by definition. Therefore, why was there a "factorial analysis" to find the optimized concentration?"

The authors responded that the NaCl concentration in the 60MME medium was determined from the factorial analysis. The only problem with this is that as written, it sounds like the 60MME medium already existed and then a new analysis was done. To clarify, I suggest the authors simply change the first instance of "60MME" on line 154 to "MME." This way, in the subsequent sentence, it becomes clear that "60MME" was defined AFTER this factorial experiment.

2. Original comment, now Line 156, Figure 3c – "60 g/L was noted to give the "highest" concentration at 2.82 g/L. Is this really significantly different from what was achieved with 80 g/L?"

The authors responded that the difference was not significant but that higher NaCl concentrations are undesirable. The authors should revise the sentence that follows to acknowledge this in the manuscript. It currently reads "Since a higher NaCl concentration failed to improve ectoine accumulation, simultaneously inhibiting the cell growth." I note that this is not a complete sentence. I suggest revising to read something like:
"The titer was comparable at higher NaCl concentration, but cell growth was inhibited."

Reviewer #3 (Remarks to the Author):

Dear Authors,

your manuscript was carefully revised, and the comments were appropriatey addressed. It can now be accepted for publication.

Reviewer #4 (Remarks to the Author):

Authors have addressed the suggestions. No more comments.

We are grateful to the **three reviewers** for their efforts to handle and review our paper. After carefully studying the comments and suggestions, and following responses have been generated (highlighted in red color):

Comments from reviewers:

Reviewer #1: unavailable.

Reviewer #2 (Remarks to the Author):
The authors have done a nice job responding to my (somewhat lengthy comments). I only have 2 points that I don't feel were adequately addressed but which can be quite easily:
1. Original comment, now Lines 153-155 – "The medium named "60MME" has 60 g/L NaCl by definition. Therefore, why was there a "factorial analysis" to find the optimized concentration?"

The authors responded that the NaCl concentration in the 60MME medium was determined from the factorial analysis. The only problem with this is that as written, it sounds like the 60MME medium already existed and then a new analysis was done. To clarify, I suggest the authors simply change the first instance of "60MME" on line 154 to "MME." This way, in the subsequent sentence, it becomes clear that "60MME" was defined AFTER this factorial experiment.

Response: Thanks for your constructive advice, revision has been done in the TEXT, line 154 as suggested.

2. Original comment, now Line 156, Figure 3c – "60 g/L was noted to give the "highest" concentration at 2.82 g/L. Is this really significantly different from what was achieved with 80 g/L?"
The authors responded that the difference was not significant but that higher NaCl concentrations are undesirable. The authors should revise the sentence that follows to acknowledge this in the manuscript. It currently reads "Since a higher NaCl concentration failed to improve ectoine accumulation, simultaneously inhibiting the cell growth." I note that this is not a complete sentence. I suggest revising to read something like:
"The titer was comparable at higher NaCl concentration, but cell growth was inhibited."

Response: Thanks for your kindly suggestion, revision has been done as suggested.
Thanks for your efforts to improve the quality of this study.

Reviewer #3 (Remarks to the Author):
Dear Authors,
your manuscript was carefully revised, and the comments were appropriately addressed.

It can now be accepted for publication.

Response: Thanks for your efforts to improve the quality of this study.

Reviewer #4 (Remarks to the Author):
Authors have addressed the suggestions. No more comments.

Response: Thanks for your efforts to improve the quality of this study.

Once again, we thank three reviewers and the editor for their precious time and energy to help improve the quality of our paper.
